# Substrate-triggered position switching of TatA and TatB during Tat transport in *Escherichia coli*

**DOI:** 10.1098/rsob.170091

**Published:** 2017-08-16

**Authors:** Johann Habersetzer, Kristoffer Moore, Jon Cherry, Grant Buchanan, Phillip J. Stansfeld, Tracy Palmer

**Affiliations:** 1 Division of Molecular Microbiology, School of Life Sciences, University of Dundee, Dundee DD1 5EH, UK; 2 Department of Biochemistry, University of Oxford, South Parks Road, Oxford OX1 3QU, UK

**Keywords:** protein transport, Tat pathway, twin-arginine signal peptide, transport mechanism

## Abstract

The twin-arginine protein transport (Tat) machinery mediates the translocation of folded proteins across the cytoplasmic membrane of prokaryotes and the thylakoid membrane of plant chloroplasts. The *Escherichia coli* Tat system comprises TatC and two additional sequence-related proteins, TatA and TatB. The active translocase is assembled on demand, with substrate-binding at a TatABC receptor complex triggering recruitment and assembly of multiple additional copies of TatA; however, the molecular interactions mediating translocase assembly are poorly understood. A ‘polar cluster’ site on TatC transmembrane (TM) helix 5 was previously identified as binding to TatB. Here, we use disulfide cross-linking and molecular modelling to identify a new binding site on TatC TM helix 6, adjacent to the polar cluster site. We demonstrate that TatA and TatB each have the capacity to bind at both TatC sites, however *in vivo* this is regulated according to the activation state of the complex. In the resting-state system, TatB binds the polar cluster site, with TatA occupying the TM helix 6 site. However when the system is activated by overproduction of a substrate, TatA and TatB switch binding sites. We propose that this substrate-triggered positional exchange is a key step in the assembly of an active Tat translocase.

## 1. Introduction

The twin-arginine protein transport (Tat) pathway operates in parallel with the general secretory (Sec) pathway to export proteins across the cytoplasmic membrane of bacteria and archaea, and the thylakoid membrane of plant chloroplasts. Tat substrates have N-terminal signal peptides containing a conserved twin-arginine motif and are transported across the membrane in a folded state driven by the protonmotive force [1–3].

The Tat machinery comprises membrane proteins from the TatA and TatC families. TatA family proteins are monotopic with an N-out transmembrane (TM) helix at their N-terminus, followed by a cytoplasmically located amphipathic helix [4,5]. Most Gram-negative bacteria, and plant thylakoids, have two functionally distinguishable TatA paralogues (TatA and TatB in bacteria) that have distinct roles in Tat transport (e.g. [6,7]). TatC is the core component of the Tat system, and forms a scaffold for the dynamic assembly of Tat complexes during protein translocation [8,9]. Tat transport is initiated by binding of the signal peptide of a Tat substrate to the Tat(A)BC receptor complex. This complex, which contains several copies of TatB and TatC, is multivalent and appears to function as an obligate oligomer [10–13]. Although the Tat(A)BC complex is stable and can interact with substrates in the absence of TatA [13,14], it is likely that *in vivo* some TatA constitutively associates with this complex, most likely in an equimolar ratio with TatB and TatC [10,15–17].

The signal peptide twin-arginine motif is recognized by the cytoplasmic surface of TatC [9,18]. The signal peptide can also bind more deeply within the receptor complex, contacting residues in the TM helix of TatB and towards the periplasmic end of TatC TM helix 5 (TM5) [18–20]. Following substrate binding, additional TatA protomers are recruited to the receptor complex dependent on the protonmotive force [16,19,21–25]. According to current models, the assembled TatA oligomer facilitates substrate translocation across the membrane either through formation of a size-variable channel or by promoting localized membrane weakening and transient bilayer disruption (see [1,2] for recent reviews).

Although high-resolution structural information is available for TatA, TatB and TatC [8,9,26–29], to date Tat complexes have only been visualized at low resolution [13,30,31]. Site-specific cross-linking has been used to map interaction interfaces between Tat components, giving results consistent with a potential binding site for TatB being located along one face of TatC TM5 [9,20,32]. One such study additionally suggested that TatB might control access of TatA to TatC [20], and a cross-linking study of the pea Tat system suggested that cross-links between Tha4 (TatA) and cpTatC TM5 were enhanced by addition of a substrate [16]. Recently, coevolution analysis independently predicted the location a TatA/TatB binding site along TM5 of TatC, pointing to a polar cluster of amino acids in *E. coli* TatC (M205, T208 and Q215) forming likely contacts with a polar side chain in TatA and TatB [15]. TatB was demonstrated to occupy this site in the resting translocase, and further experiments with alanine-substituted polar cluster variants suggested that TatA and TatB might differentially occupy the same TatC TM5 site at different stages of Tat transport [15].

In this work, we have undertaken an *in vivo* disulfide cross-linking study to explore the interaction of TatC with TatA and TatB in the absence of a bound substrate and when a substrate is likely to be bound. Our studies identify two binding sites for each protein. The first of these, at TatC TM5, is occupied by TatB under resting conditions, consistent with the studies described above. We identified an additional binding site located at TatC TM6 which we show is occupied by TatA in the resting state. Combining the cross-linking data with evolutionary coupling and molecular modelling allowed us to predict the precise position of the entire TatA TM helix, which was shown by molecular dynamics simulation to be stable in this site, and was confirmed by further targeted cross-linking experiments. We go on to show that in the presence of over-expressed Tat substrate TatA and TatB move positions to occupy each other's binding sites, and we therefore propose that signal peptide-triggered position switching of TatA and TatB is a critical step in driving the assembly of an active Tat translocase.

## 2. Results

### 2.1. The TatB TM helix is positioned close to TM5 of TatC at the polar cluster site under resting conditions

**Figure 1.**
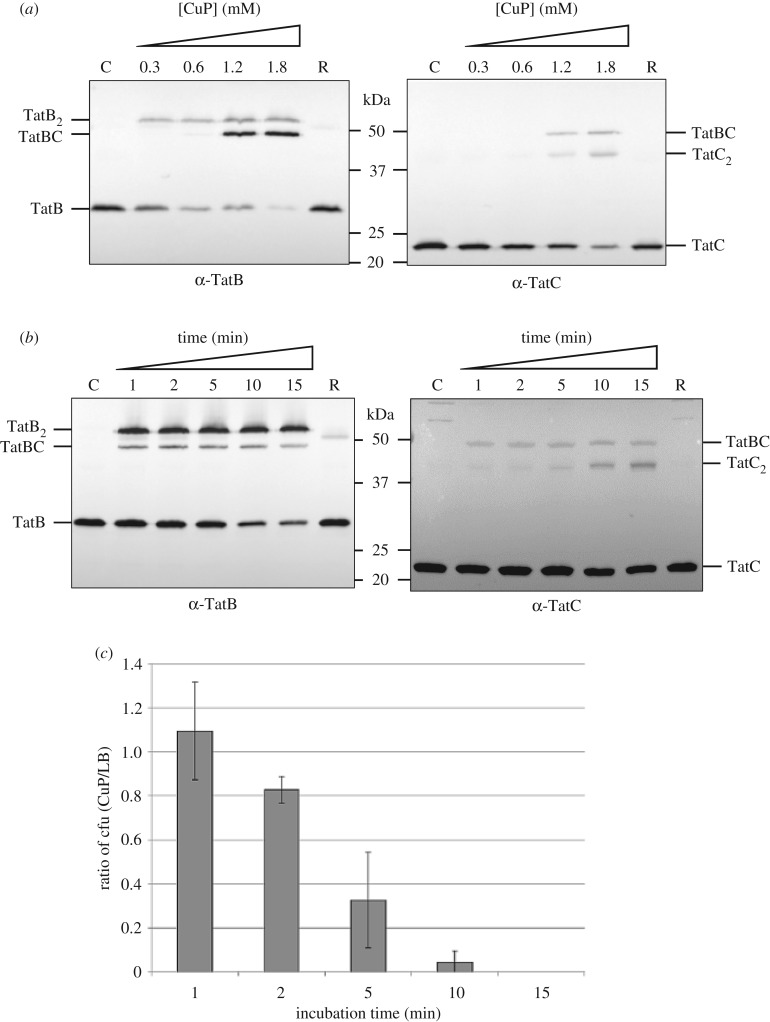
Development of an *in vivo* disulfide cross-linking protocol. Cells of strain MC4100ΔBC (Δ*tatBC*) harbouring plasmid p101C*BC producing TatB^L9C^ alongside TatC^M205C^ were incubated with either LB medium (control, C), or LB supplemented with 10 mM DTT (reduced; R) or (*a*) the indicated concentrations of CuP for 15 min or (*b*) with 1.8 mM CuP for 1–15 min. The reaction was quenched by addition of 8 mM NEM/12 mM EDTA, membranes were prepared and proteins were separated by SDS-PAGE (10% polyacrylamide). Cross-linked products were visualized by immunoblotting using anti-TatB_FL_ or anti-TatC antibodies, as indicated. (*c*) Aliquots of cells from the oxidized and control samples in (*b*) were spread on LB plates containing chloramphenicol and the number of colonies enumerated following growth at 37°C for 24 h. The *y*-axis shows the ratio of the number of colony forming units (cfu) obtained after incubation with 1.8 mM CuP compared to the number after incubation in LB medium only; *n* = 3 biological replicates, error bars are ±s.d.

Prior disulfide cross-linking studies between *E. coli* TatB and TatC used isolated membrane fractions harbouring elevated copies of Tat components. Under these conditions an initial contact site between TatB^L9C^ and TatC^M205C^ was identified [32], which was subsequently extended to reveal further contacts between Cys residues introduced into the TM helix of TatB and into TM5 of TatC [9]. To explore whether the same contact sites were detectable *in vivo*, we developed a protocol for disulfide cross-linking in intact cells using the TatB^L9C^ –TatC^M205C^ cross-link. In these experiments, the Cys-substituted variants of TatB and TatC were produced from the low copy number plasmid p101C*BC, which expresses *tatBC* at approximately chromosomal level [21], in a strain lacking chromosomal *tatBC*. An initial titration with the oxidant copper phenanthroline (CuP) revealed that a TatBC cross-link was detectable when CuP was used at 1.2 and 1.8 mM (figure 1*a*). We also noted that TatB and TatC homodimers were formed through the introduced Cys residues after incubation with CuP, as reported previously [9,32]. Next, using 1.8 mM CuP, we undertook a time course from 1 to 15 min and examined the formation of the TatBC heterodimer and the survival of cells during this period. Figure 1*b* shows a TatBC heterodimer was detected at all time points, including the earliest time point tested; however, incubation times with CuP in excess of 1 min saw a significant reduction in the recovery of cells (figure 1*c*). We therefore chose to use a 1 min incubation with 1.8 mM CuP for all subsequent cross-linking analysis.

**Figure 2.**
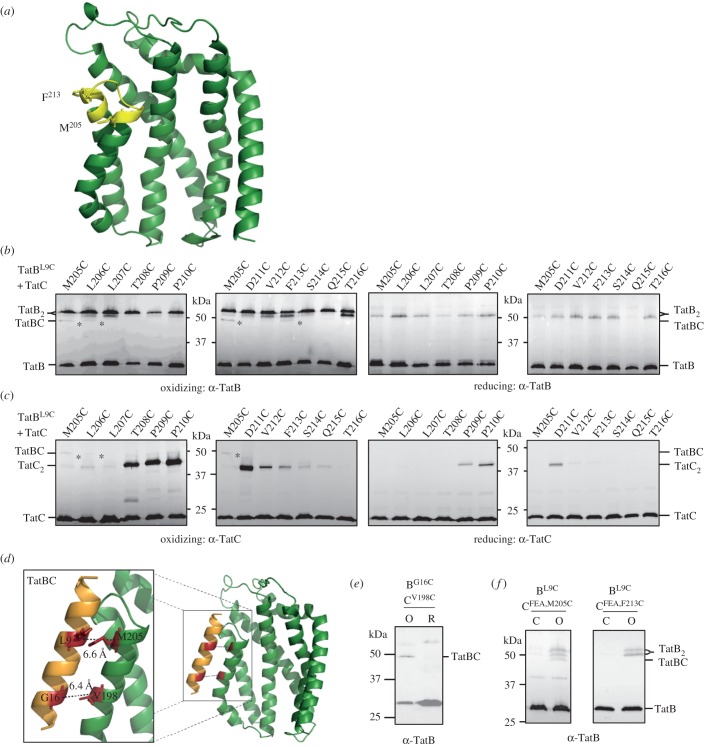
TatB^L9C^ interacts with TatC^M205C^ *in vivo*. (*a*) Homology model of *E. coli* TatC showing positions of the residues used for disulfide cross-linking analysis in yellow. The side-chains of M205 and F213 are indicated. (*b*,*c*) Western blot analysis (separated on 10% polyacrylamide gels) of membranes from *E. coli* strain MC4100ΔBC producing TatB^L9C^ alongside the indicated Cys substitutions in TatC (from plasmid p101C*BC) following exposure of whole cells to 1.8 mM CuP (oxidizing) or 10 mM DTT (reducing) for 1 min. Cross-linked products were visualized by immunoblotting using (*b*) an anti-TatB peptide antibody or (*c*) an anti-TatC antibody. The asterisks indicate likely TatBC cross-links. (*d*) Structural model of TatB interacting with TatC at the polar cluster site (adapted from [15]). The backbone distances between TatB^L9^/TatC^M205^ and TatB^G16^/TatC^V198^ are shown. (*e*) Whole cells of strain MC4100ΔBC producing TatB^L9C^ alongside TatC^F94A,E103,/M205C^ or TatC^F94A/E103A/F213C^ from plasmid p101C*BC (annotated TatC^FEA,M205C^ or TatC^FEA,F213C^, respectively) were left untreated (C) or incubated for 1 min with 1.8 mM CuP (O) as indicated. Following membrane preparation, cross-links were detected with an anti-TatB peptide antibody.

Next we introduced Cys residues into a scanning region of TatC from residue 205 in TM5, through the periplasmic P3 loop as far as residue 216 in TM6 (figure 2*a*). Figure S1 in the electronic supplementary material shows that when each of these TatC Cys substitutions was co-produced with TatB^L9C^, cells were able to grow in the presence of 2% SDS. This indicates successful export of Tat substrates AmiA and AmiC [33] and therefore that the Cys substitutions did not abolish Tat transport activity. Following incubation of cells producing each of these variants with CuP, a TatBC heterodimer was primarily detected between TatB^L9C^ and TatC^M205C^ (figure 2*b*,*c*). A faint TatBC heterodimer band was also seen between TatB^L9C^ and TatC^L206C^, and a fainter one between TatB^L9C^ and TatC^F213C^ that was only detected with the anti-TatB antibody (indicated with asterisks on figure 2*b*). It should be noted that the TatB antiserum used in this scanning experiment is a polyclonal anti-peptide antibody that primarily recognizes the C-terminal 15 amino acids of TatB and detects the TatB homodimer as a doublet band (electronic supplementary material, figure S2) for reasons that are unclear.

Alcock *et al.* [15] identified a binding site for TatA/TatB close to the polar cluster of residues M205, T208 and Q215 in TatC. Molecular dynamics simulations (MDS) indicated that TatB E8 may hydrogen bond with both T208 and Q215 when bound at this site. We were unable to explore this directly by disulfide cross-linking because a Cys substitution at TatB^E8^ abolished Tat activity when expressed from plasmid p101C*BC (electronic supplementary material, figure S1*a*). This is consistent with the loss of activity noted for a TatB^E8A^ substitution, which resulted in destabilization of the TatB–TatC interaction [15]. However, molecular modelling indicates that when TatB interacts with TatC via the polar cluster, L9 of TatB may be positioned within 6.6 Å of TatC^M205^ (backbone distances; figure 2*d*). We therefore conclude that the disulfide cross-link formed between TatB^L9C^ and TatC^M205C^ arises from interaction of TatB at the TatC polar cluster site. To confirm this we undertook disulfide cross-linking between TatB^G16C^ and TatC^V198C^ (figure 2*d*), which are one of the most highly covarying pairs of residues at the polar cluster site [15]. Figure 2*e* shows that, as expected, a cross-link is formed between these two cysteine residues when cells were oxidized. These results give full support to the binding mode of TatB described previously [15].

Experiments using a variant of TatC that is unable to bind signal peptides (TatC^F94A,E103A^) led to the conclusion that the interaction of TatB at the TatC polar cluster site occurred when the Tat system was at rest [15]. Figure 2*f* shows that in agreement with this, introduction of these same TatC substitutions did not abolish the TatB^L9C^ and TatC^M205C^ cross-link. Thus TatB occupies the polar cluster binding site under resting conditions *in vivo*.

### 2.2. In the resting Tat system TatA interacts at a distinct site on TatC close to TM6

Next we used a similar approach to determine whether we could detect *in vivo* interactions between TatA and TatC. Initially, Cys-substituted variants of TatA and TatC were produced alongside TatB under control of the *lac* promoter from plasmid pQE60 and expressed in strain DADE-P [34] that lacks chromosomally encoded *tatABC*/*tatE* and which harbours the *pcnB1* allele to limit plasmid copy number to 1–2 per cell [35]. Cys substitutions were introduced at L9, L10 and I11 of TatA and these were tested with the same Cys-scanning region from residues 205–216 of TatC.

**Figure 3.**
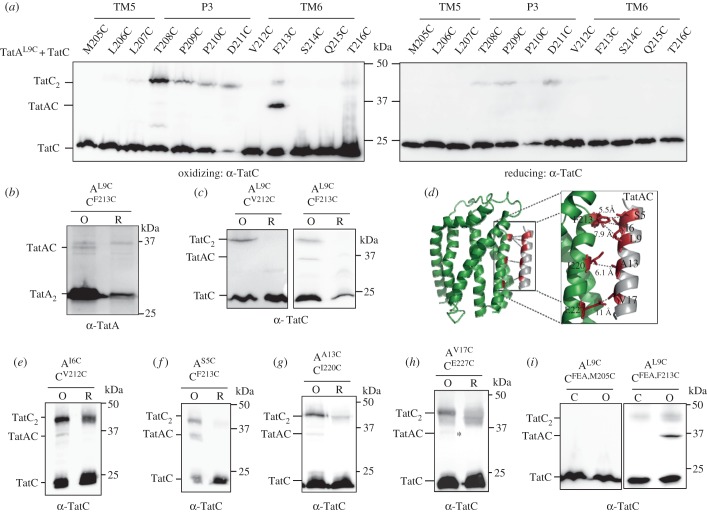
TatA^L9C^ interacts with TatC^F213C^ *in vivo*. (*a*,*e*,*g,h*) Western blot analysis (separated on 12.5% polyacrylamide gels) of whole cells of *E. coli* strain DADE-P producing the indicated Cys substitutions in TatA and TatC (and wild-type TatB, from plasmid pUNITATCC4) following exposure to 1.8 mM CuP (oxidizing) or 10 mM DTT (reducing) for 1 min. Cross-linked products were visualized by immunoblotting using anti-TatC antibodies. The asterisk in (*h*) indicates a faint TatAC cross-link. (*b*) The TatA^L9C^–TatC^F213C^ oxidized (O) and reduced (R) samples from (*a*) were separately probed with an anti-TatA antibody (note that the TatA monomer that is in large excess has been run off the bottom of the gel). (*c,f*) Cells of strain DADE harbouring plasmid pTAT101 producing (*c*) TatA^L9C^ and wild-type TatB along with either TatC^V212C^ or TatC^F213C^, or (*f*) TatA^S5C^, wild-type TatB and TatC^F213C^, as indicated, were incubated with 1.8 mM CuP (O) or 10 mM DTT (R) for 1 min. (*d*) Structural model of TatA interacting with TatC at the TatA constitutive binding site. The backbone distances between TatA^S5/L9^/TatC^F213^, TatA^I6^/TatC^V212^, TatA^A13^/TatC^I220^ and TatA^V17^/TatC^E227^ are shown. (*i*) Cells of strain DADE producing TatA^L9C^ and wild-type TatB alongside TatC^F94A,E103A,M205C^ or TatC^F94A,E103A,F213C^ (annotated TatC^FEA,M205C^ or TatC^FEA,F213C^, respectively) from pTAT101 were left untreated (C) or incubated with 1.8 mM CuP (O) for 1 min. For (*c,d*), following quenching, membranes were prepared, samples separated by SDS-PAGE (12.5% polyacrylamide) and immunoblotted using an anti-TatC antibody.

First we confirmed that Tat activity was not abolished following introduction of any of these substitutions by showing that each pair of Cys-substituted proteins was able to support growth of DADE-P in the presence of 2% SDS (electronic supplementary material, figure S1*b*,*c*). Subsequently we undertook cross-linking analysis *in vivo* using the same protocol as that used for TatB–TatC cross-linking. Under the conditions tested, no cross-links were detected between TatA and the TatC polar cluster residue M205C (figure 3*a*; electronic supplementary material, figure S3), indicating that TatA is not present at this site. Instead, a band of the expected size for a TatA–TatC heterodimer was detected under oxidizing conditions in cells co-producing TatA^L9C^ and TatC^F213C^. This was confirmed as a cross-link between TatC and TatA because it was also cross-reactive with an anti-TatA antibody (figure 3*b*). A similar band was also detectable under oxidizing conditions when TatA^L9C^ and TatC^F213C^ were produced alongside wild-type TatB at much lower levels from plasmid pTAT101 (figure 3*c*). Scanning analysis using TatA^L10C^ revealed a faint cross-link with TatC^V212C^ following oxidation (electronic supplementary material, figure S3*a*), and TatAI11C gave detectable cross-links with TatC^V212C^ and TatC^F213C^ (electronic supplementary material, figure S3*b*).

**Figure 4.**
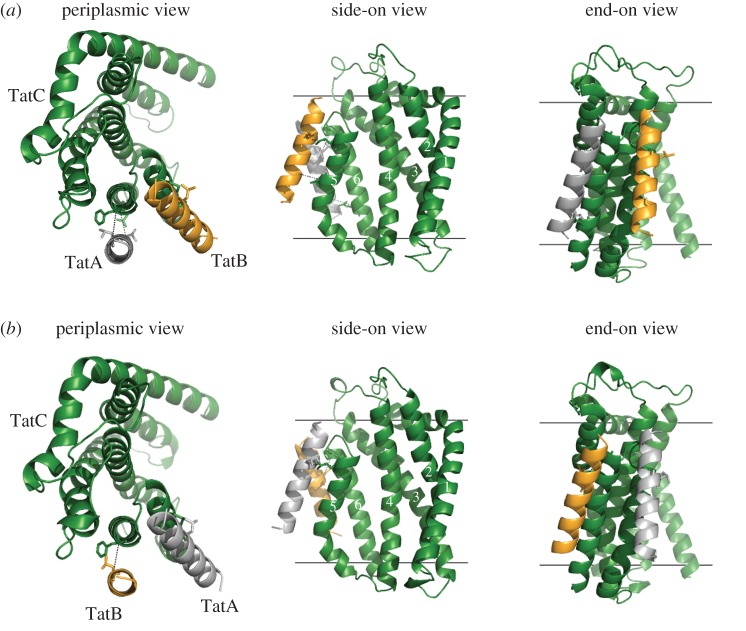
Models of the TatABC trimer in the resting and activated state. Three views of (*a*) the resting-state TatABC complex and (*b*) the substrate-activated TatABC complex. TatA is shown in silver, TatB gold and TatC green. Note that in (*b*) the substrate signal peptide is not shown as it is currently unclear precisely where it binds in the activated state.

Taken together, the absence of a TatA cross-link at the TatC polar cluster site along with clear cross-links between TatA^L9C/L11C^ and the N-terminal end of TatC TM6 suggests that TatA occupies a distinct binding site. Co-evolutionary analysis identified a weak evolutionary coupling between TatA/B residue 17 and TatC residue 227 (*E. coli* numbering), that was much lower than the primary contacts identified previously [15]. Guided by this and the TatA/TatC cross-links identified above we were able to dock TatA into a binding site that lies adjacent to the polar cluster site (figure 3*d* and figure 4*a*). Atomistic molecular dynamic simulations suggested that TatA was stable in this site (electronic supplementary material, figures S4A, S5) and together with the modelling predicted further contacts between TatA and TatC including S5–F213, I6–V212 and A13–I220. To confirm this, we constructed cysteine substitutions at each of these predicted pairs, and were able to detect oxidant-induced TatA–TatC heterodimers at each of these positions (figure 3*e–g*). We were also able to detect a very faint heterodimeric cross-link between TatA^V17C^ and TatC^E227C^ (figure 3*h*). We conclude that TatA occupies a binding site that is distinct from, but adjacent to, the polar cluster site.

TatA associates with TatC in two different modes. One of these is constitutive, whereas the second is induced in the presence of substrate and is associated with Tat transport [15–17,20,24]. Substrate-induced assembly of TatA can be abolished by alanine substitution of two key residues of TatC, F94 and E103, which constitute the signal peptide binding site [21]. To determine whether TatA interaction at this newly identified site was independent of substrate-binding we introduced the F94A and E103A substitutions into TatC^F213C^ and probed for interaction with TatA^L9C^. Figure 3*i* shows that the TatAC cross-link was still strongly detected, and therefore arises due to substrate-independent binding of TatA.

Collectively these results demonstrate that there are two binding sites for TatA family proteins on *E. coli* TatC and that under resting conditions TatB occupies the TM5/polar cluster site while TatA is bound at the TM6 site. Molecular modelling suggests that both of these sites can be simultaneously occupied on a single TatC (figure 4*a*). Atomistic molecular dynamics suggest that this ternary complex is stable as a TatA_1_B_1_C_1_ heterotrimer (electronic supplementary material, figure S4*a*, S5), with stability further increased for a TatA_3_B_3_C_3_ oligomer (electronic supplementary material, figure S4*b*, S6), and structural stability plots indicate that the secondary structure in the starting models was preserved (electronic supplementary material, figure S7*a*,*c*).

### 2.3. TatA and TatB are each capable of occupying both binding sites

**Figure 5.**
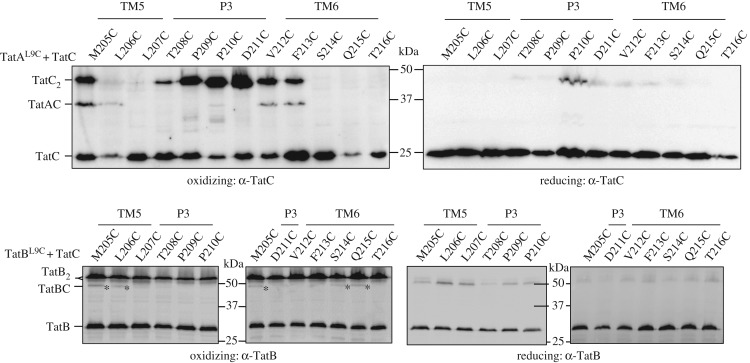
TatA and TatB can each occupy both binding sites on TatC. (*a*) Western blot analysis (separated on 12.5% polyacrylamide gels) of whole cells of *E. coli* strain DADE-P producing TatA^L9C^ alongside the indicated Cys substitutions in TatC (in the absence of TatB, from plasmid pUNITATCC4ΔB) following exposure to 1.8 mM CuP (oxidizing) or 10 mM DTT (reducing) for 1 min. Cross-linked products were visualized by immunoblotting using anti-TatC antibodies. (*b*) Western blot analysis (separated on 10% polyacrylamide gels) of membranes from *E. coli* strain DADE producing TatB^L9C^ alongside the indicated Cys substitutions in TatC (from plasmid p101C*BC) following exposure of whole cells to 1.8 mM CuP (oxidizing) or 10 mM DTT (reducing) for 1 min. Cross-linked products were visualized by immunoblotting using an anti-TatB peptide antibody. The asterisks indicate likely TatBC cross-links.

Having defined two binding sites on TatC for a TatA/B TM helix, and identified diagnostic cysteine cross-linking positions for each site, we next asked the question whether TatA and TatB were each capable of occupying both binding sites if they were the only TatA family protein present. Figure 5*a* shows that in the absence of TatB, a Cys substitution at TatA^L9^ still disulfide cross-links with TatC^F213C^, indicating that it occupies the TM6 binding site. However, additional cross-links were now also detected between TatA^L9C^ and TatC^M205C/L206C^ which are adjacent to the polar cluster site. This finding indicates that TatA is capable of binding in both sites.

When TatA was absent, cross-links of TatB^L9C^ to TatC^M205C^ and TatC^L206C^ were still detected, indicating occupancy at the polar cluster site, but additional cross-links were also now detected between TatB^L9C^ and TatC^S214C/Q215C^, showing that TatB can also bind at the TM6 binding site if this site is vacant (figure 5*b*). Thus we conclude that each protein is able to occupy both binding sites.

### 2.4. TatA and TatB switch binding sites in the presence of a Tat substrate

**Figure 6.**
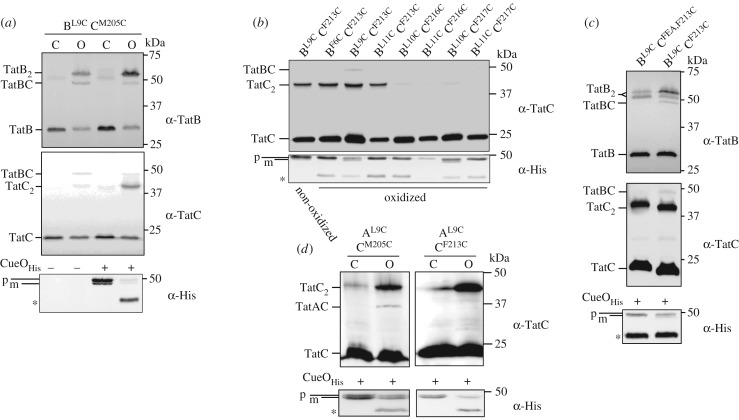
TatA and TatB cross-linking patterns are altered in the presence of an overproduced Tat substrate. (*a*) Strain MC4100ΔBC harbouring plasmid p101C*BC producing TatB^L9C^ alongside TatC^M205C^ and plasmid pQE80-CueO where indicated, were left untreated (control, C), or incubated with 1.8 mM CuP for 1 min (O). Membrane fractions were prepared, separated by SDS-PAGE (10% polyacrylamide) and immunoblotted with anti-TatB_FL_, anti-TatC as indicated. An aliquot of the soluble fraction following membrane preparation was retained and analysed by immunoblotting with an anti-Histag antibody. (*b*) Strain MC4100ΔBC producing the indicated Cys variants of TatB and TatC from plasmid p101C*BC and his-tagged CueO from pQE80-CueO were incubated with 1.8 mM CuP for 1 min. Following quenching, membrane fractions were separated by SDS-PAGE (10% polyacrylamide) and immunoblotted with an anti-TatC antibody. A non-oxidized sample of membranes harbouring TatB^L9C^–TatC^MF213C^ is shown in the left-most lane. An aliquot of the soluble fraction from each sample was retained and analysed by immunoblotting with an anti-Histag antibody. (*c*) Whole cells of strain MC4100ΔBC producing TatB^L9C^ alongside TatC^F213C^ or TatC^F94A,E103A,M205C^ (annotated TatC^FEA,M205C^) from plasmid p101C*BC, and his-tagged CueO (from pQE80-CueO) were incubated for 1 min with 1.8 mM CuP. Following membrane preparation, cross-links were detected with an anti-TatB peptide antibody or an anti-TatC antibody. An aliquot of the soluble fraction from each sample was retained and analysed by immunoblotting with an anti-Histag antibody. (*d*) Strain DADE harbouring plasmid pTAT101 producing wild-type TatB, TatA^L9C^ and either TatC^M205C^ or TatC^F213C^ along with plasmid pQE80-CueO were left untreated (control, C), or incubated with 1.8 mM CuP for 1 min (O). Membrane fractions were separated by SDS-PAGE (12.5% polyacrylamide) and immunoblotted with an anti-TatC antibody. An aliquot of the soluble fraction from each sample was retained and analysed by immunoblotting with an anti-Histag antibody. p: precursor, m: mature forms of substrate CueO-His * indicates an oxidation product of CueO.

We next addressed whether differential occupancy of TatA and TatB at these binding sites was functionally related to Tat transport. To this end we undertook disulfide cross-linking analysis in the presence of an overproduced Tat substrate, CueO. We focused initially on the interaction of TatB^L9C^ and TatC^M205C^ that reports on the presence of TatB at the polar cluster site. When CueO was overproduced, the level of cross-linking between TatB^L9C^ and TatC^M205C^ appeared to diminish and the level of TatB and TatC homodimers to increase compared to those seen in the presence of endogenous substrate proteins (figure 6*a*). It should be noted, in agreement with this, that substrate-induced TatC homodimerization through M205C has previously been observed [11]. This finding is consistent with the idea that there is substrate-induced movement of TatB^L9^ away from TatC^M205^. To determine whether TatB may now occupy the second TatA/TatB binding site, we probed for cross-links between TatB^L6C^, TatB^L9C^, TatB^L10C^ or TatB^L11C^ and Cys substitutions at positions 213, 216 or 217 of TatC, each in the presence of overproduced CueO. Figure 6*b* shows that a cross-link could be detected between TatB^L9C^ and TatC^F213C^. Substrate-induced reduction in cross-linking between TatB^L9C^ and TatC^M205C^ and a concomitant increase in the TatB^L9C^ and TatC^F213C^ cross-link is also shown for a different plasmid construct in the electronic supplementary material, figure S8*a*.

It was noted in figure 2 that in the presence of endogenous substrate a very faint cross-link between TatB^L9C^ and TatC^F213C^ could be detected with the anti-TatB antibody (but not the anti-TatC antibody), and this can also be seen in the electronic supplementary material, figure S8A. The observation that this cross-link was abolished when substrate binding to the TatBC complex was prevented by introduction of the TatC^F94A,E103A^ substitutions (figure 2*f*), even when substrate was overproduced (figure 6*c*), strongly suggests that docking of a substrate to the receptor complex triggers the movement of TatB into the TM6 binding site.

As TatB can occupy the TM6 site in the presence of overproduced substrate, it should be accompanied by loss of TatA from this binding site. Figure 6*d* shows, in agreement with this, that a cross-link between TatA^L9C^ and TatC^F213C^ could no longer be detected when CueO was overproduced. A further repeat of this experiment shown in the electronic supplementary material, figure S8*a* confirms the reduction in TatA^L9C^–TatC^F213C^ cross-linking when CueO was overproduced. Thus, overproducing a Tat substrate results in a change of occupancy at the TM6 binding site, with TatB replacing TatA. To determine whether TatA was now present at the polar cluster binding site, cross-linking between TatA^L9C^ and TatC^M205C^ was analysed in the presence of CueO. Figure 6*d* indicates that overproduced substrate induced the formation of a cross-link between these residues in TatA and TatC and that therefore TatA is able to occupy the polar cluster site in the presence of substrate. An increase in the level of the TatA^L9C^–TatC^M205C^ heterodimeric cross-link with increasing substrate production is also shown in the electronic supplementary material, figure S8*b,c*. It should be noted, however, that at the same level of exposure as the other TatAC and TatBC cross-linking analyses, the TatA^L9C^–TatC^M205C^ heterodimer was too weak to be detected (electronic supplementary material, figure S8*a*). The reason for this is not clear but it is possible that the occupancy and/or conformation of TatA at the polar cluster might be influenced by the further polymerization of TatA molecules during the assembly of the transport-associated TatA oligomer.

Molecular dynamic simulations have previously shown that TatA can stably interact with TatC through the polar cluster site [15]. A similar analysis indicates that TatB can bind at the TatA constitutive site and that it can stably occupy that site when TatA is bound at the polar cluster site (electronic supplementary material, figures S9, S10). A model for the substrate-activated state of the TatABC complex is shown in figure 4*b*.

## 3. Discussion

In this study, we have used disulfide cross-linking to probe the interaction of TatA and TatB with TatC under resting conditions and in the presence of an over-expressed substrate. Our results have delineated two binding sites for these proteins. One of these—the ‘polar cluster’ site—has been identified previously and involves key interactions between a polar side chain at position 8 of TatA/B and a patch of conserved residues, M205, T208 and Q215, in TatC [15]. The second site lies adjacent to the polar cluster site, at TM6 of TatC. Experiments where TatA or TatB were present individually as the sole TatA/B family protein indicated that each of these proteins was capable of occupying both sites. However, locking the Tat system into the resting state through the introduction of substitutions in TatC that prevent signal peptide binding demonstrated that TatB occupies the polar cluster site under these circumstances, as proposed previously [15], with TatA occupying the newly identified site. Modelling and MDS suggested that interaction of TatA with the TM6 binding site was stable and that both of these sites can be simultaneously occupied on one TatC protein. This adjacent positioning of TatA and TatB is supported by the detection of TatA–TatB cross-links when a photocrosslinker is introduced into the N-terminal region of TatB [20].

**Figure 7.**
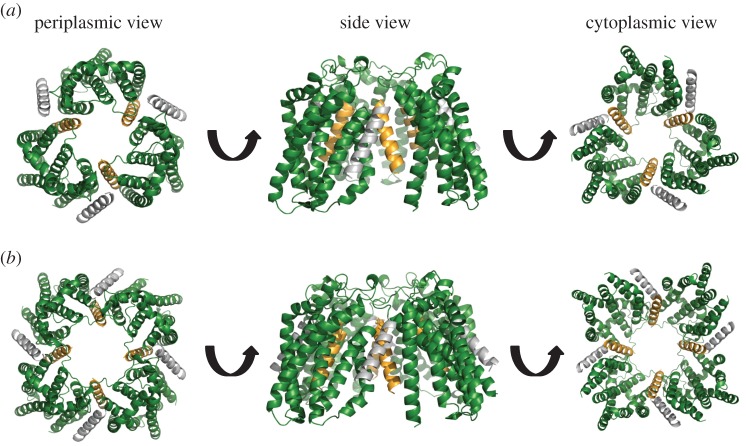
Models of the multimeric resting-state TatABC complex. Models based on (*a*) three or (*b*) four heterotrimers. Modified from Alcock *et al.* [15].

Recently, a molecular model of the multivalent resting-state TatBC complex was built by docking TatBC protomers together using evolutionary couplings between TatC proteins and cross-links between the TatC TM1 and the TatB TM helix [15,20]. Updated models for the resting TatABC complex containing either three or four copies of the heterotrimer can be seen in figure 7. TatA can be readily accommodated into the complex with minimal adjustment, slotting into a groove that is present at the outside of the complex. This peripheral binding of TatA probably explains why a TatBC complex can be stably purified when TatA is absent (e.g. [36,37]) and may potentially account for findings that TatA is variably shed from the TatBC complex during purification in detergent solution [10,38].

When a Tat substrate protein is overproduced, we show that the level of TatA cross-linking at the TM6 site is reduced and that TatA becomes detectable by cross-linking at the polar cluster site. This is accompanied by a reduction in the level of TatB cross-linking at the polar cluster site and the appearance of TatB–TatC cross-links at the TatA constitutive site. In agreement with this, substrate-dependent contacts between the chloroplast TatA orthologue Tha4 and cpTatC at the equivalent polar cluster site have also been observed [16]. Position-switching of TatA and TatB is probably triggered by signal peptide binding at the complex. In the deep-binding mode, contacts have been detected between the signal peptide h-region and both the TatB TM helix and TatC TM5, close to the polar cluster site [16,19,20]. While current findings cannot distinguish whether it is TatA or TatB that makes the initial movement, based on previous findings we speculate it is TatB. It has been shown by several genetic studies that suppressors of inactive twin-arginine signal peptides or a defective TatC signal peptide binding site locate primarily to the TM helix of TatB. No such suppressors have yet been identified in TatA. Cross-linking analysis indicated that at least some of these TatB suppressor variants caused rearrangement at the polar cluster site, probably by decreasing TatB binding affinity at this site and/or increasing binding affinity for the constitutive site [39–41]. It has been shown that Tat signal peptides are sequestered within a cavity comprising TatB and TatC, which could potentially correspond to the central cavity seen in the modelled TatBC/TatABC complexes (figure 4*b* and [15,20]). Note that the polar cluster site is adjacent to the lumen of this cavity and, accordingly, docking of the signal peptide close to this region may cause conformational rearrangements that drive TatB from the polar cluster site into the TatA constitutive site.

In addition to changes in TatA and TatB cross-linking patterns, we note that signal peptide binding also resulted in the formation of TatB homodimers through L9C, and TatC homodimers mediated through TatC M205C or through F213C. The formation of a substrate-induced TatC M205C homodimer has been observed previously and taken to report on the activated state of the Tat translocase [11,39]. The head-to-tail arrangement of TatC in the resting-state model of TatABC complex positions neighbouring TatC M205 residues 25 Å away from each other, and F213 residues even further apart, a distance that is too great for disulfide bond formation through Cys sidechains at these positions. This strongly suggests that there must be a significant conformational change in the TatABC complex upon substrate binding to bring TatC protomers into a tail-to-tail organization. Opening up of the complex in this way would then allow TatA to access the vacated polar cluster site. It should be noted that the concave face of TatC has been implicated in the nucleation of the transport-active TatA oligomer [9,16]. Binding of a TatA molecule at the polar cluster site places it adjacent to the concave face where it could potentially initiate polymerization of further TatA molecules.

In conclusion, we have defined two binding sites for TatA family proteins within the TatABC complex and have demonstrated differential occupancy of TatA and TatB at these sites during different stages of Tat transport. These findings help to explain a long-standing observation that overproduction of TatB relative to TatA and TatC inactivates the Tat system [7], because simultaneous occupancy of TatB (which is normally present 20-fold less than TatA [31,42]) in both binding sites would be expected to block progression through the transport cycle.

## 4. Material and methods

### 4.1. Strains and plasmids and growth conditions

All strains used for cross-linking analysis are derived from MC4100 (F^−^, [*araD139*]*_B/r_*, Δ(*argF-lac*)*U169*, *λ^−^*, *e14-*, *flhD5301*, Δ(*fruK-yeiR*)*725*(*fruA25*), *relA1*, *rpsL150*(Str^R^), *rbsR22*, Δ(*fimB-fimE*)*632*(*::IS1*), *deoC1*—[43]). MC4100ΔBC (as MC4100, Δ*tatBC*—[21]), DADE (as MC4100, Δ*tatABCD* Δ*tatE—*[44]) and DADE-P (as DADE*, pcnB1 zad-*981*::*Tn*10*d (Kan^r^)—[34]) were used where indicated in the figure legends. Strain JM109 (F′ *traD36 proA^+^B^+^ lacI^q^* Δ(*lacZ*)*M15/* Δ(*lac-proABI glnV44 e14^−^ gyrA96 recA1 relA1 endA1 thi hsdR17*) was used for cloning purposes.

All plasmids used in this study are listed in the electronic supplementary material, table S1. Plasmid pUNITATCC4 encodes TatA, TatB and cysteine-less TatC in plasmid pQE60. Production of the encoded proteins is driven by the phage *T5* promoter which is constitutively active in strains deleted for *lacI*, such as MC4100 derivatives. Plasmid pUNITATCC4ΔB (producing TatA and TatC) was derived from pQEA(DB)C [45] by excising DNA covering the wild-type allele of *tatC* through digestion with *Xho*I and *Bam*HI and replacement with a Cys-less *tatC* allele amplified using oligonucleotides TatBdeldownXho [45] and TatCBam [34] with pUNITATCC4 as template. Plasmid pTAT101 codes for TatA, TatB and TatC on a low copy number vector and produces these proteins at approximately four times chromosomal level [32]. Plasmid p101C*BC expresses *tatBC* at approximately chromosomal level and has been described previously [21]. p101C*BC Cys-less was designed as follows: a *tatBC* allele where all of four Cys codons of *tatC* had been mutated to Ala codons was amplified from pTat101 Cys-less [11] using primers BamHI-TatB-F and SpHI-TatC-R (electronic supplementary material, table S2), introducing a *Sph*I site at the 3′-end of *tatC*. The PCR product was digested with *Bam*HI/*Sph*I and cloned into similarly digested p101C*BC. All point mutations in plasmids were introduced by Quickchange site-directed mutagenesis (Stratagene) using the primers listed in the electronic supplementary material, table S2. Plasmid pQE80-CueO expresses *E. coli* CueO with a C-terminal his6 tag and has been described previously [46]. Plasmid pTGS encodes GFP fused to the TorA signal sequence and a C-terminal SsrA tag [47].

Phenotypic growth in the presence of 2% SDS was assessed by culturing strains of interest in LB medium containing appropriate antibiotics until an OD_600_ of 1 was reached, after which 5 µl aliquots of culture were spotted onto agar plates containing LB or LB supplemented with 2% SDS and appropriate antibiotics. Plates were incubated at 37°C for 16 h after which they were photographed. Antibiotics were used at the following concentrations: chloramphenicol (25 µg ml^−1^), kanamycin (50 µg ml^−1^) and ampicillin (125 µg ml^−1^).

### 4.2. *In vivo* disulfide cross-linking experiments

For Tat proteins produced at close to native level (from pTAT101 and p101C*BC), the appropriate *E. coli* strain/plasmid combination was cultured overnight in LB medium containing appropriate antibiotics. Cells were diluted 1 : 100 into fresh LB medium supplemented with appropriate antibiotics and cultured aerobically until an OD_600_ of 0.3 was reached. For the CuP titration experiment, six 25 ml aliquots were withdrawn and each supplemented with fresh LB medium to a final OD_600_ of 0.15. The first aliquot was left untreated (control), the second one was supplemented with 10 mM DTT (reducing) and the remainder were incubated with 0.3, 0.6, 1.2 or 1.8 mM CuP (oxidizing). Cells were incubated for 15 min at 37°C with agitation, then harvested, resuspended in 1 ml 20 mM Tris–HCl, pH 7.5, 200 mM NaCl, 12 mM EDTA, 8 mM *N*-ethylmaleimide and incubated at 37°C for 10 min to quench free sulfhydryls. The cell suspension was supplemented with protease inhibitor cocktail (Roche) and disrupted by sonication. Unbroken cells were removed by centrifugation (10 000*g* for 5 min at 4°C) and the supernatant ultracentrifuged (200 000*g* for 30 min at 4°C). The membrane pellet was resuspended in 70 µl 1× Laemmli buffer lacking β-mercaptoethanol (BioRad). For the time course experiment, when subcultured cells reached OD_600_ of 0.3, seven 25 ml aliquots were withdrawn and supplemented with fresh LB medium to a final OD_600_ of 0.15. One aliquot was left untreated, one was supplemented with 10 mM DTT and the remainder incubated with 1.8 mM CuP for 1, 2, 5, 10 or 15 min at 37°C with agitation. The reactions were quenched for 10 min as before and a small aliquot of cells from each sample was withdrawn, serially diluted and spread on LB plates supplemented with appropriate antibiotics to assess viability. Membrane samples were prepared from the remainder of the cells and treated as described above. For all other experiments, when cells reached OD_600_ 0.3, three separate 25 ml aliquots were withdrawn and supplemented with fresh medium to OD_600_ of 0.15. One aliquot was left untreated, the second supplemented with 10 mM DTT and the third incubated with 1.8 mM CuP for 1 min at 37°C with agitation. The reactions were quenched and membrane samples prepared as described before. When experiments were performed in the presence of overproduced CueO, cells additionally harboured pQE80-CueO and IPTG was included in the initial subculture. IPTG was added to a final concentration of 1 mM unless otherwise stated.

For Tat proteins produced at higher copy from plasmid pUNITATCC4, overnight cultures of DADE-P harbouring pUNITATCC4 were subcultured at 1 : 100 to inoculate fresh LB containing appropriate antibiotics. When cells reached OD_600_ of 0.3, three 2.5 ml aliquots were withdrawn and made up to 5 ml with fresh LB to a final OD_600_ of 0.15. These aliquots were treated and quenched as described above after which the cells were harvested at 16 000*g* for 1 min and resuspended in 40 µl of 1× Laemmli buffer lacking β-mercaptoethanol (BioRad). Where experiments were undertaken in the presence of TorAss-GFP-SsrA, cells were subcultured until an OD_600_ of 0.25 was reached, after which l-arabinose at concentrations between 0.001 and 0.01% was added and cells incubated aerobically for a further 20 min before being diluted to a final OD_600_ of 0.15 and treated as described above. Although high concentrations of l-arabinose are toxic to *E. coli* MC4100 strain derivatives, control growth experiments confirmed that at the concentrations used here there was no loss of cell viability.

For analysis, sodium dodecyl sulfate polyacrylamide gel electrophoresis (SDS-PAGE) was performed using Tris-glycine gels [48]. In total, 20 µl of sample was analysed in each case. Following electrophoresis, proteins were transferred to nitrocellulose membrane (I-blot^®^ system, Life Technologies). TatA and TatC were identified using the polyclonal antibodies previously described [11,31]. Two different TatB antisera were used. One of these was raised against full-length TatB [31] and in this study is annotated as TatB_FL_. The second was raised against two peptides of *E. coli* TatB 69–84 and TatB 156–171, and was then affinity purified with peptide 156–171 [15] and in this study is referred to as a TatB peptide antibody. Abcam Anti-6X His tag^®^ antibody [GT359] (HRP conjugate) was purchased (catalogue number ab184607), and an HRP-conjugated goat anti-rabbit antibody (BioRad, catalogue number 170–6515) was used as secondary antibody for the TatA, TatB and TatC antisera. Cross-reacting bands were visualized after incubation with Clarity™ Western ECL Blotting Substrate (BioRad) using a CCCD camera (GeneGnome XRQ, Syngene).

### 4.3. Molecular modelling and simulations

Molecular modelling was carried out as described previously [15]. All images were generated using Pymol (The PyMol Molecular Graphics System, Version 1.8, Schrödinger, LLC). Multimers were built using TatA–TatC/TatB–TatC disulfide cross-links as unambiguous constraints for docking using HADDOCK [49]. In all of these experiments, TatA is modelled from residues G2 to G21, TatB from residues F2 to G21 and TatC from residues T11 to F235.

All MDS were performed using GROMACS v. 5.1.2 [50]. The Martini 2.2 force field [51] was used to run initial 1 µs coarse-grained (CG) MDS to permit the assembly and equilibration of 1-palmitoyl, 2-oleoyl phosphatidylglycerol : 1-palmitoyl, 2-oleoyl phosphatidylethanolamine bilayers around the TatABC complexes at a 1 : 3 molar ratio [52]. CG molecular systems were converted to atomistic detail using CG2AT [53], with Alchembed used to remove any unfavourable steric contacts between protein and lipid [54]. The heterotrimeric atomistic systems equate to a total size of approximately 80 000 atoms and box dimensions in the region of 125 × 125 × 100 Å^3^, while the heterononameric systems comprised approximately 115 000 atoms, with box dimensions in the region of 100 × 100 × 100 Å^3^. The systems were equilibrated for 1 ns with the protein restrained before three repeats of 100 ns of unrestrained atomistic MDS, for each configuration of the molecular system (see below), using the Gromos53a6 force field [55]. Molecular systems were neutralized with a 150 mM concentration of NaCl.

All simulations were executed at 37°C, with protein, lipids and solvent separately coupled to an external bath, using the velocity-rescale thermostat [56]. Pressure was maintained at 1 bar, with a semi-isotropic compressibility of 4 × 10^−5^ using the Parrinello–Rahman barostat [57]. All bonds were constrained with the LINCS algorithm [58]. Electrostatics was measured using the Particle Mesh Ewald method [59], while a cut-off was used for Lennard–Jones parameters, with a Verlet cut-off scheme to permit GPU calculation of non-bonded contacts. Simulations were performed with an integration time step of 2 fs. Analysis was performed using GROMACS tools and locally written python and perl scripts.

## Supplementary Material

Supplementary Figures and Tables

## Data accessibility

The datasets supporting this article have been uploaded as part of the electronic supplementary material.
