# Supplementary material for: Substrate-triggered position switching of TatA and TatB during Tat transport in Escherichia coli
Source: Open Biol. 2017 Aug 16;7(8):170091. doi: 10.1098/rsob.170091 (PMC5577447; doi:10.1098/rsob.170091)
Supplement: Supplementary Figures and Tables [file rsob170091supp1.pdf]

Substrate-triggered position-switching of TatA and TatB in the *Escherichia coli* Tat protein export pathway.

Johann Habersetzer[1]*, Kristoffer Moore[1]* Jon Cherry[1], Grant Buchanan[1], Phillip Stansfeld[2] and Tracy Palmer[1$]

Supplementary Information

Plasmid	Relevant genotype	Source
pUNITATCC4	pQE60, encoding TatA, TatB and TatC (all four Cys of TatC substituted for Ala)	[34]
pUNI4 AI6C, CV212C	pUNITATCC4, $TatA^{I6C}$, $TatC^{V212C}$	This study
pUNI4 AL9C, CM205C	pUNITATCC4, $TatA^{L9C}$, $TatC^{M205C}$	This study
pUNI4 AL9C, CL206C	pUNITATCC4, $TatA^{L9C}$, $TatC^{L206C}$	This study
pUNI4 AL9C, CL207C	pUNITATCC4, $TatA^{L9C}$, $TatC^{L207C}$	This study
pUNI4 AL9C, CT208C	pUNITATCC4, $TatA^{L9C}$, $TatC^{T208C}$	This study
pUNI4 AL9C, CP209C	pUNITATCC4, $TatA^{L9C}$, $TatC^{P209C}$	This study
pUNI4 AL9C, CP210C	pUNITATCC4, $TatA^{L9C}$, $TatC^{P210C}$	This study
pUNI4 AL9C, CD211C	pUNITATCC4, $TatA^{L9C}$, $TatC^{D211C}$	This study
pUNI4 AL9C, CV212C	pUNITATCC4, $TatA^{L9C}$, $TatC^{V212C}$	This study
pUNI4 AL9C, CF213C	pUNITATCC4, $TatA^{L9C}$, $TatC^{F213C}$	This study
pUNI4 AL9C, CS214C	pUNITATCC4, $TatA^{L9C}$, $TatC^{S214C}$	This study
pUNI4 AL9C, CQ215C	pUNITATCC4, $TatA^{L9C}$, $TatC^{Q215C}$	This study
pUNI4 AL9C, CT216C	pUNITATCC4, $TatA^{L9C}$, $TatC^{T216C}$	This study
pUNI4 AL10C, CM205C	pUNITATCC4, $TatA^{L10C}$, $TatC^{M205C}$	This study
pUNI4 AL10C, CL206C	pUNITATCC4, $TatA^{L10C}$, $TatC^{L206C}$	This study
pUNI4 AL10C, CL207C	pUNITATCC4, $TatA^{L10C}$, $TatC^{L207C}$	This study
pUNI4 AL10C, CT208C	pUNITATCC4, $TatA^{L10C}$, $TatC^{T208C}$	This study
pUNI4 AL10C, CP209C	pUNITATCC4, $TatA^{L10C}$, $TatC^{P209C}$	This study
pUNI4 AL10C, CP210C	pUNITATCC4, $TatA^{L10C}$, $TatC^{P210C}$	This study
pUNI4 AL10C, CD211C	pUNITATCC4, $TatA^{L10C}$, $TatC^{D211C}$	This study
pUNI4 AL10C, CV212C	pUNITATCC4, $TatA^{L10C}$, $TatC^{V212C}$	This study
pUNI4 AL10C, CF213C	pUNITATCC4, $TatA^{L10C}$, $TatC^{F213C}$	This study
pUNI4 AL10C, CS214C	pUNITATCC4, $TatA^{L10C}$, $TatC^{S214C}$	This study
pUNI4 AL10C, CQ215C	pUNITATCC4, $TatA^{L10C}$, $TatC^{Q215C}$	This study
pUNI4 AL10C, CT216C	pUNITATCC4, $TatA^{L10C}$, $TatC^{T216C}$	This study
pUNI4 AL11C, CM205C	pUNITATCC4, $TatA^{L11C}$, $TatC^{M205C}$	This study
pUNI4 AL11C, CL206C	pUNITATCC4, $TatA^{L11C}$, $TatC^{L206C}$	This study
pUNI4 AL11C, CL207C	pUNITATCC4, $TatA^{L11C}$, $TatC^{L207C}$	This study
pUNI4 AL11C, CT208C	pUNITATCC4, $TatA^{L11C}$, $TatC^{T208C}$	This study
pUNI4 AL11C, CP209C	pUNITATCC4, $TatA^{L11C}$, $TatC^{P209C}$	This study
pUNI4 AL11C, CP210C	pUNITATCC4, $TatA^{L11C}$, $TatC^{P210C}$	This study
pUNI4 AL11C, CD211C	pUNITATCC4, $TatA^{L11C}$, $TatC^{D211C}$	This study
pUNI4 AL11C, CV212C	pUNITATCC4, $TatA^{L11C}$, $TatC^{V212C}$	This study
pUNI4 AL11C, CF213C	pUNITATCC4, $TatA^{L11C}$, $TatC^{F213C}$	This study
pUNI4 AL11C, CS214C	pUNITATCC4, $TatA^{L11C}$, $TatC^{S214C}$	This study
pUNI4 AL11C, CQ215C	pUNITATCC4, $TatA^{L11C}$, $TatC^{Q215C}$	This study
pUNI4 AL11C, CT216C	pUNITATCC4, $TatA^{L11C}$, $TatC^{T216C}$	This study
pUNI4 AA13C, CI220C	pUNITATCC4, $TatA^{A13C}$, $TatC^{I220C}$	This study
pUNI4 AV17C, CE227C	pUNITATCC4, $TatA^{V17C}$, $TatC^{E227C}$	This study
pUNITATCC4 ΔB	As pUNITATCC4, in frame deletion of *tatB*	This study

pUNI4ΔB AL9C, CM205C	pUNITATCC4ΔB, TatA^{L9C}, TatC^{M205C}	This study
pUNI4ΔB AL9C, CL206C	pUNITATCC4ΔB, TatA L9C, TatC^{L206C}	This study
pUNI4ΔB AL9C, CL207C	pUNITATCC4ΔB, TatA L9C, TatC^{L207C}	This study
pUNI4ΔB AL9C, CT208C	pUNITATCC4ΔB, TatA^{L9C}, TatC^{T208C}	This study
pUNI4ΔB AL9C, CP209C	pUNITATCC4ΔB, TatA^{L9C}, TatC^{P209C}	This study
pUNI4ΔB AL9C, CP210C	pUNITATCC4ΔB, TatA^{L9C}, TatC^{P210C}	This study
pUNI4ΔB AL9C, CD211C	pUNITATCC4ΔB, TatA^{L9C}, TatC^{D211C}	This study
pUNI4ΔB AL9C, CV212C	pUNITATCC4ΔB, TatA^{L9C}, TatC^{V212C}	This study
pUNI4ΔB AL9C, CF213C	pUNITATCC4ΔB, TatA^{L9C}, TatC^{F213C}	This study
pUNI4ΔB AL9C, CS214C	pUNITATCC4ΔB, TatA^{L9C}, TatC^{S214C}	This study
pUNI4ΔB AL9C, CQ215C	pUNITATCC4ΔB, TatA^{L9C}, TatC^{Q215C}	This study
pUNI4ΔB AL9C, CT216C	pUNITATCC4ΔB, TatA^{L9C}, TatC^{T216C}	This study
pTAT101	Low copy number vector producing TatABC under control of *tat* promoter. Kan^{r}	[32]
pTAT101 Cys-less	As pTat101, all four Cys codons of *tatC* substituted for Ala codons)	[11]
p101CC4 AS5C, CF213C	pTat101 Cys-less, TatA^{S5C}, TatC^{F213C}	This study
p101CC4 AL9C, CM205C	pTat101 Cys-less, TatA^{L9C}, TatC^{M205C}	This study
p101CC4 AL9C, CV212C	pTat101 Cys-less, TatA^{L9C}, TatC^{V212C}	This study
p101CC4 AL9C, CF213C	pTat101 Cys-less, TatA^{L9C}, TatC^{F213C}	This study
P101CC4 BL9C, CM205C	pTat101 Cys-less, TatB^{L9C}, TatC^{M205C}	This study
p101CC4 BL9C, CF213C	pTat101 Cys-less, TatB^{L9C}, TatC^{F213C}	This study
p101CC4 AL9C, CFEA,M205C	pTat101 Cys-less, TatA^{L9C}, TatC^{F94A,E103A,M205C}	This study
p101CC4 AL9C, CFEA,F213C	pTat101 Cys-less, TatA^{L9C}, TatC^{F94A,E103A,F213C}	This study
p101C*BC	pTH19Cr derivative. Expression of *tatBC* from the *tatA* promoter with a modified RBS.	[21
p101C*BC Cys-less	As p101C*BC, producing Cys-less variant of TatC	This study
p101C*BF6C, CF213C	As p101C*BC Cys-less, TatB^{F6C}, TatC^{F213C}	This study
p101C*BE8C, CM205C	As p101C*BC Cys-less, TatB^{E8C}, TatC^{M205C}	This study
p101C*BL9C	As p101C*BC Cys-less, TatB^{L9C}	This study
p101C*BL9C, CM205C	As p101C*BC Cys-less, TatB^{L9C}, TatC^{M205C}	This study
p101C*BL9C, CL206C	As p101C*BC Cys-less, TatB^{L9C}, TatC^{L206C}	This study
p101C*BL9C, CL207C	As p101C*BC Cys-less, TatB^{L9C}, TatC^{L207C}	This study
p101C*BL9C, CT208C	As p101C*BC Cys-less, TatB^{L9C}, TatC^{T208C}	This study
p101C*BL9C, CP209C	As p101C*BC Cys-less, TatB^{L9C}, TatC^{P209C}	This study
p101C*BL9C, CP210C	As p101C*BC Cys-less, TatB^{L9C}, TatC^{P210C}	This study
p101C*BL9C, CD211C	As p101C*BC Cys-less, TatB^{L9C}, TatC^{D211C}	This study
p101C*BL9C, CV212C	As p101C*BC Cys-less, TatB^{L9C}, TatC^{V212C}	This study
p101C*BL9C, CF213C	As p101C*BC Cys-less, TatB^{L9C}, TatC^{F213C}	This study
p101C*BL9C, CS214C	As p101C*BC Cys-less, TatB^{L9C}, TatC^{S214C}	This study
p101C*BL9C, CQ215C	As p101C*BC Cys-less, TatB^{L9C}, TatC^{Q215C}	This study
p101C*BL9C, CT216C	As p101C*BC Cys-less, TatB^{L9C}, TatC^{T216C}	This study

p101C*BL10C, CL216C	As p101C*BC Cys-less, $TatB^{L10C}$, $TatC^{L216C}$	This study
p101C*BL10C, CF217C	As p101C*BC Cys-less, $TatB^{L10C}$, $TatC^{F217C}$	This study
p101C*BL11C, CF213C	As p101C*BC Cys-less, $TatB^{L11C}$, $TatC^{F213C}$	This study
p101C*BL11C, CL216C	As p101C*BC Cys-less, $TatB^{L11C}$, $TatC^{L216C}$	This study
p101C*BL11C, CF217C	As p101C*BC Cys-less, $TatB^{L11C}$, $TatC^{F217C}$	This study
p101C*BG16C, CV198C	As p101C*BC Cys-less, $TatB^{G16C}$, $TatC^{V198C}$	This study
p101C*BL9C, CFEA,M205C	As p101C*BC Cys-less, $TatB^{L9C}$, $TatC^{F94A,E103A,M205C}$	This study
p101C*BL9C, CFEA,F213C	As p101C*BC Cys-less, $TatB^{L9C}$, $TatC^{F94A,E103A,F213C}$	This study
pQE80-CueO	As pQE80, encoding *E. coli* CueO with C-terminal his_6-tag.	[21]
pTGS	pBAD33-based vector encoding TorAss-GFP-SsrA	[47]

Table S1. Plasmids used in this study.

Primer name	Sequence (5’-3’)
TatAS5CF	ATGGGTGGTATCTGTATTTGGCAGTTATTGATT
TatAS5CR	TAACAATAACTGCCAAATACAGATACCACCCAT
TatAI6CF	ATGGGTGGTATCAGTTGTTGGCAGTTATTGATT
TatAI6CR	AATCAATAACTGCCAACAACTGATACCACCCAT
TatAL9CF	AATTTGGCAGTTATTGTGTATTGCCGTCATGTT
TatAL9CR	AACATGACGGCAATACACAATAACTGCCAAATT
TatAL10CF	AGTATTTGGCAGTTATGCATTATTGCCGTCATC
TatAL10CR	GATGACGGCAATAATGCATAACTGCCAAATACT
TatAI11CF	AACGATGACGGCAATACACAATAACTGCCAAAT
TatAI11CR	AATTTGGCAGTTATTGTGTATTGCCGTCATGTT
TatAA13CF	GCAGTTATTGATTATTTGCGTCATCGTTGTACTG
TatAA13CR	CAGTACAACGATGACGCAAATAATCAATAACTG
TatAV17CF	GATTATTGCCGTCATCGTTTGTCTGCTTTTTGGCACC
TatAV17CR	GGTGCCAAAAAGCAGACAAACGATGACGGCAATAATC
TatB_F6C_F	GTGTTTGATATCGGTTGTAGCGAACTGCTATTG
TatB_F6C_R	CAATAGCAGTTCGCTACAACCGATATCAAACAC
TatB_E8C_F	GATATCGGTTTTAGCTGCCTGCTATTGGTGTTC
TatB_E8C_R	GAACACCAATAGCAGGCAGCTAAAACCGATATC
TatB_L9C_F	ATCGGTTTTAGCGAATGTCTATTGGTGTTCATC
TatB_L9C_R	GATGAACACCAATAGACATTCGCTAAAACCGAT
TatB_L10C_F	GGTTTTAGCGAACTGTGTTTGGTGTTCATCATC
TatB_L10C_R	GATGATGAACACCAAACACAGTTCGCTAAAACC
TatB_L11C_F	TTTAGCGAACTGCTATGTGTGTTCATCATCGGC
TatB_L11C_R	GCCGATGATGAACACACATAGCAGTTCGCTAAA
TatB_G16C_F	AACGGTAGCGGGCTTGTTTCGCGCGTTGCGTTC
TatB_G16C_R	GAACGCAACGCGCGAAACAAGCCCGCTACCGTT
TatC_V198C_F	CGCCCGTATGTGCTGTGTGGTGCATTCGTTGTC
TatC_V198C_R	GACAACGAATGCACCACACAGCACATACGGGCG
TatC_M205C_F	GCATTCGTTGTCGGGTGTTTGCTGACGCCGCCG
TatC_M205C_R	CGGCGGCGTCAGCAAACACCCGACAACGAATGC
TatC_L206C_F	TTCGTTGTCGGGATGTGTCTGACGCCGCCGGAT
TatC_L206C_R	ATCCGGCGGCGTCAGACACATCCCGACAACGAA
TatC_L207C_F	GTTGTCGGGATGTTGTGTACGCCGCCGGATGTC
TatC_L207C R	GACATCCGGCGGCGTACACAACATCCCGACAAC
TatB_T208C_F	GTCGGGATGTTGCTGTGCCCGCCGGATGTCTTC
TatB_T208C_R	GAAGACATCCGGCGGGCACAGCAACATCCCGAC
TatB_P209C_F	GGGATGTTGCTGACGTGCCCGGATGTCTTCTCG
TatB_P209C_R	CGAGAAGACATCCGGGCACGTCAGCAACATCCC
TatB_P210C_F	ATGTTGCTGACGCCGTGTGATGTCTTCTCGCAA
TatB_P210C_R	TTGCGAGAAGACATCACACGGCGTCAGCAACAT
TatC_D211C_F	TTGCTGACGCCGCCGTGTGTCTTCTCGCAAACG
TatC_D211C_R	CGTTTGCGAGAAGACACACGGCGGCGTCAGCAA
TatB_V212C_F	CTGACGCCGCCGGATTGTTTCTCGCAAACGCTG

TatB_V212C_R	CAGCGTTTGCGAGAAACAATCCGGCGGCGTCAG
TatC_F213C_F	CGCCGGATGTCTGCTCGCAAACGCTG
TatC_F213C_R	CAGCGTTTGCGAGCAGACATCCGGCG
TatC_S214C_F	GCCGGATGTCTTCTGCCAAACGCTGTTGG
TatC_S214C_R	CCAACAGCGTTTGGCAGAAGACATCCGGC
TatC_Q215C_F	CGGATGTCTTCTCGTGCACGCTGTTGGCGATC
TatC_Q215C_R	GATCGCCAACAGCGTGCACGAGAAGACATCCG
TatC_T216C_F	GATGTCTTCTCGCAATGCCTGTTGGCGATCCCG
TatC_T216C_R	CGGGATCGCCAACAGGCATTGCGAGAAGACATC
TatC_I220C_F	CAAACGCTGTTGGCGTGCCCGATGTACGCTCTG
TatC_I220C_R	CAGAGCGTACATCGGGCACGCCAACAGCGTTTG
TatC_E227C_F	GATGTACGCTCTGTTTTGCATCGGTGTCTTCTTC
TatC_E227C_R	GAAGAAGACACCGATGCAAAACAGAGCGTACATC
TatC_F94A_F	TATCAGGTGTGGGCAGCTATCGCCCCAGCGCTG
TatC_F94A_R	CAGCGCTGGGGCGATAGCTGCCCACACCTGATA
TatC_E103A_F	TGTATAAGCATGCGCGTCGCCTGGTG
TatC_E103A_R	CCACCAGGCGACGCGCATGCTTATAC
BamHI-TatB_F	CCGGATCCGTGTTTGATATCGGTTTTAGCGAACTGC
SphI_TatC_R	GTCCGCATGCTTATTCTTCAGTTTTTTCGCTTTCTGC

Table S2. Oligonucleotides used in this study

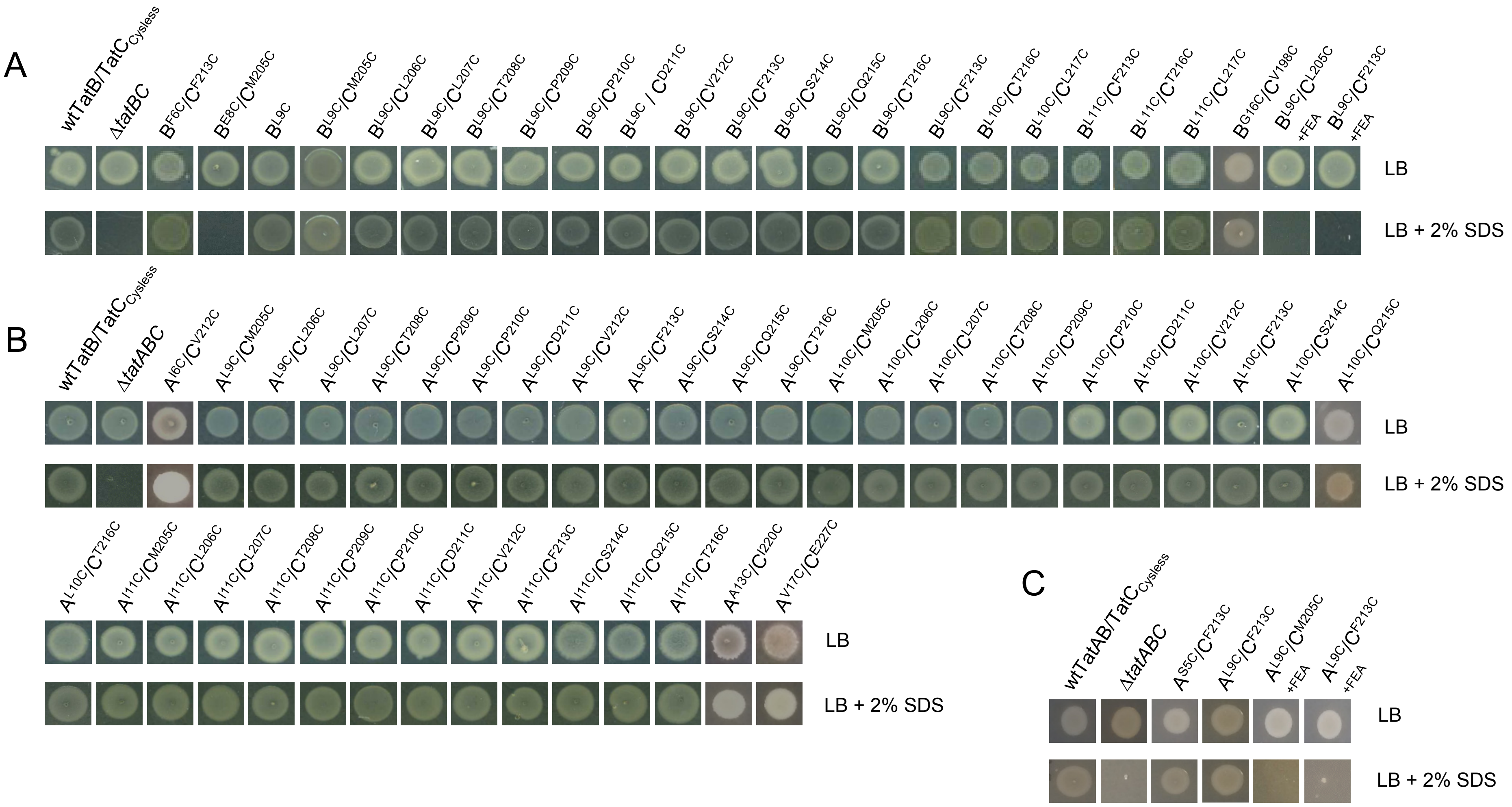

Figure S1. Phenotypic analysis of Tat activity in cells harbouring cysteine substitutions of TatA, TatB and TatC. A. TatB/TatC mutant pairs. Strain MC4100ΔBC harbouring either an empty vector (Δ*tatBC*) or plasmid p101C*BC encoding the indicated TatB and TatC variants alongside wild type TatA was spotted on LB medium or LB medium containing 2% SDS. **B.** and **C.** TatA/TatC mutant pairs **B.** Strain DADE-P harboring either an empty vector (Δ*tatABC*) or plasmid pUNITATCC4 encoding the indicated TatA and TatC variants alongside wild type TatB was spotted on LB medium or LB medium containing 2% SDS. **C**. Strain DADE harbouring either an empty vector (Δ*tatABC*) or plasmid pTAT101 encoding the indicated TatA and TatC variants alongside wild type TatB was spotted on LB medium or LB medium containing 2% SDS. In each case an 8μl aliquot of each strain/plasmid combination following aerobic growth to an OD_{600} of 1.0 was spotted and incubated for 16 hr at 37°C prior to photographing.

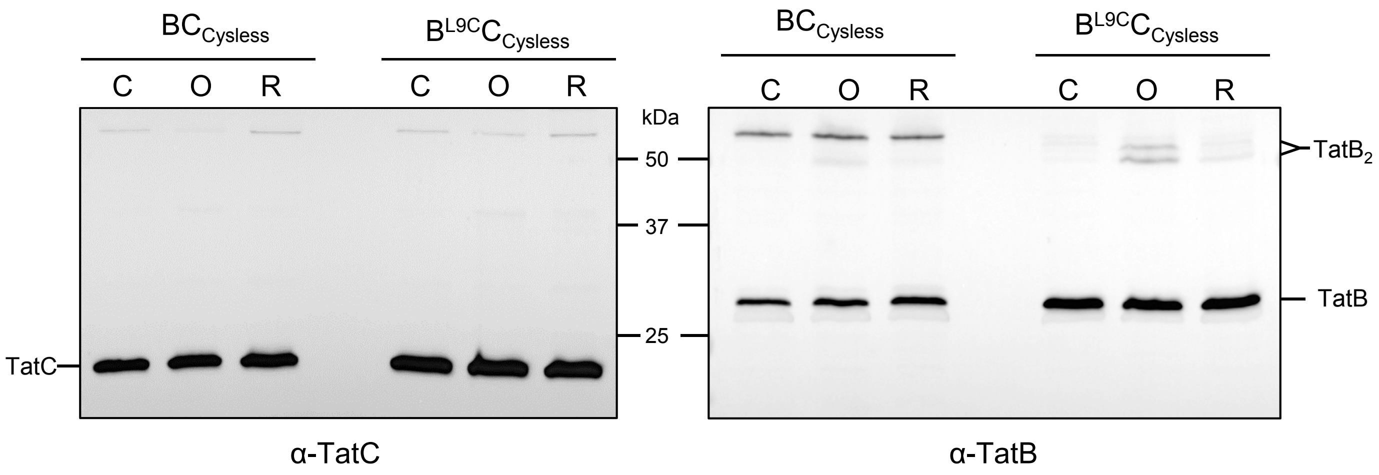

Figure S2. The TatB homodimer is recognized as a doublet band by the TatB anti-peptide antibody. Western blot analysis of membranes from *E. coli* strain MC4100ΔBC producing Cys-less TatC along with either native (Cys-less) TatB or TatBL9C, as indicated, from plasmid p101C*BC. Whole cells were either exposed to 1.8mM CuP (oxidising; O) or 10 mM DTT (reducing; R) for 1 min, or left untreated (control; C). TatC and TatB were visualised by immunoblotting using anti-TatC or anti-TatB peptide antibodies.

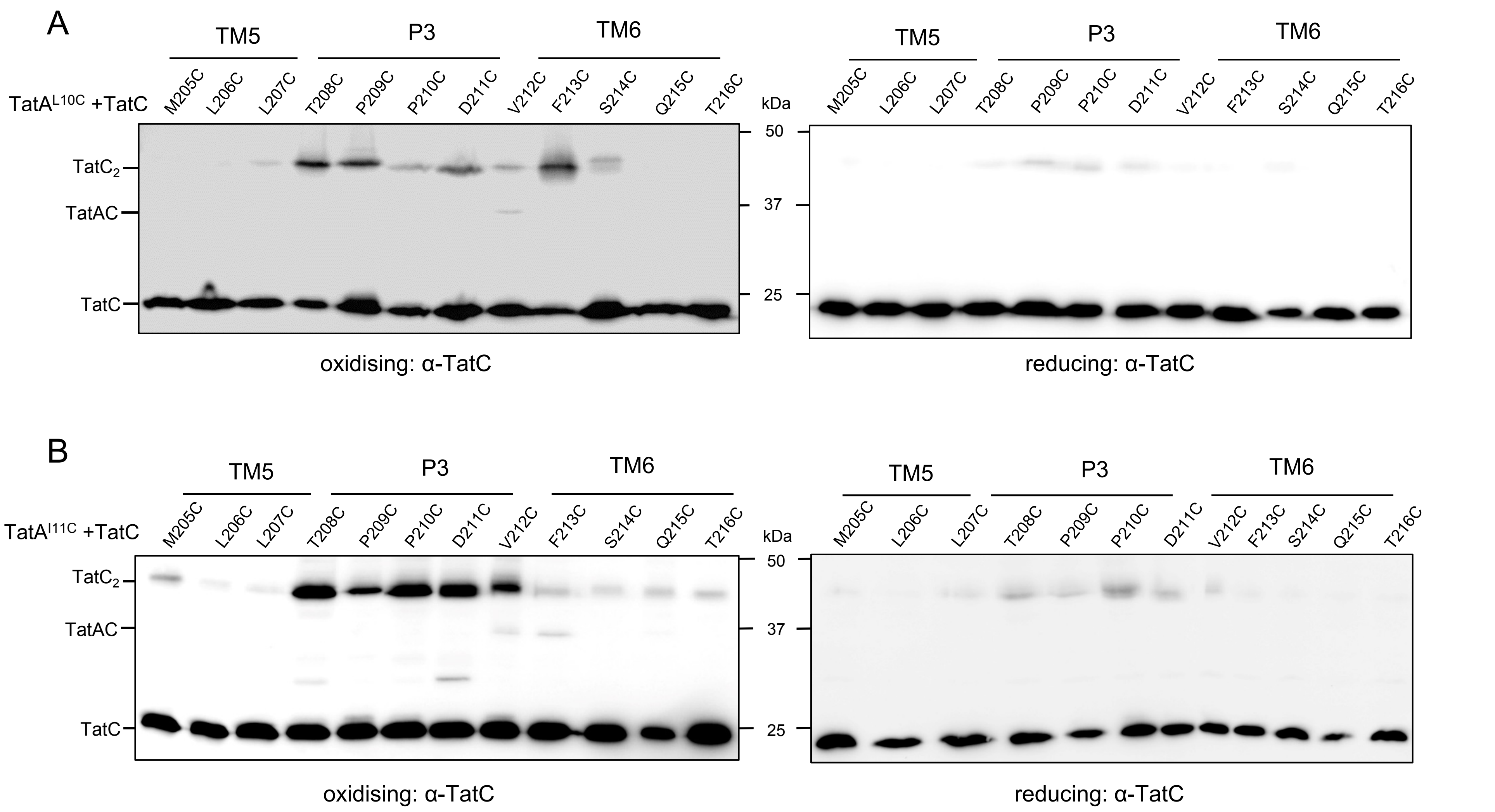

Figure S3. TatA^{L10C} interacts with TatC^{V212C} and TatA^{I11C} interacts with TatC$^{V212C/F213C}$ *in vivo*. A. and **B.** Western blot analysis (separated on 12.5% polyacrylamide gels) of whole cells of *E. coli* strain DADE-P producing **A.** TatA^{L10C} or **B.** TatA^{I11C} alongside the indicated Cys substitutions in TatC (and wild type TatB, from plasmid pUNITATCC4) following exposure to 1.8mM CuP (oxidizing) or 10 mM DTT (reducing) for 1 min. Crosslinked products were visualized by immunoblotting using anti-TatC antibodies.

A

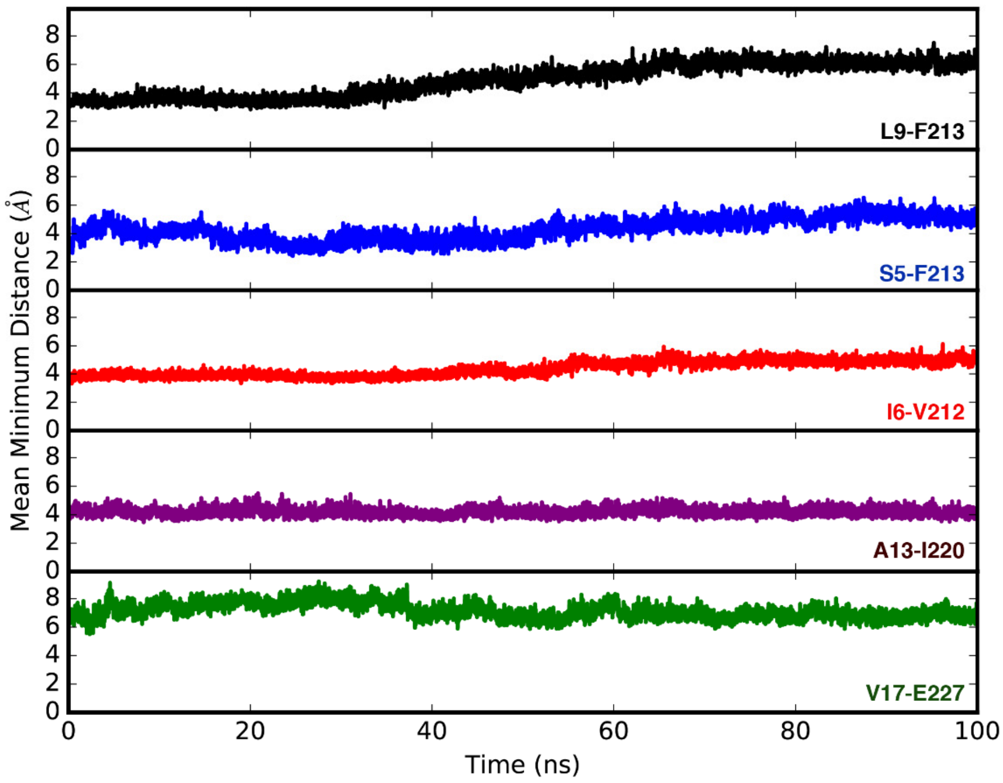

B

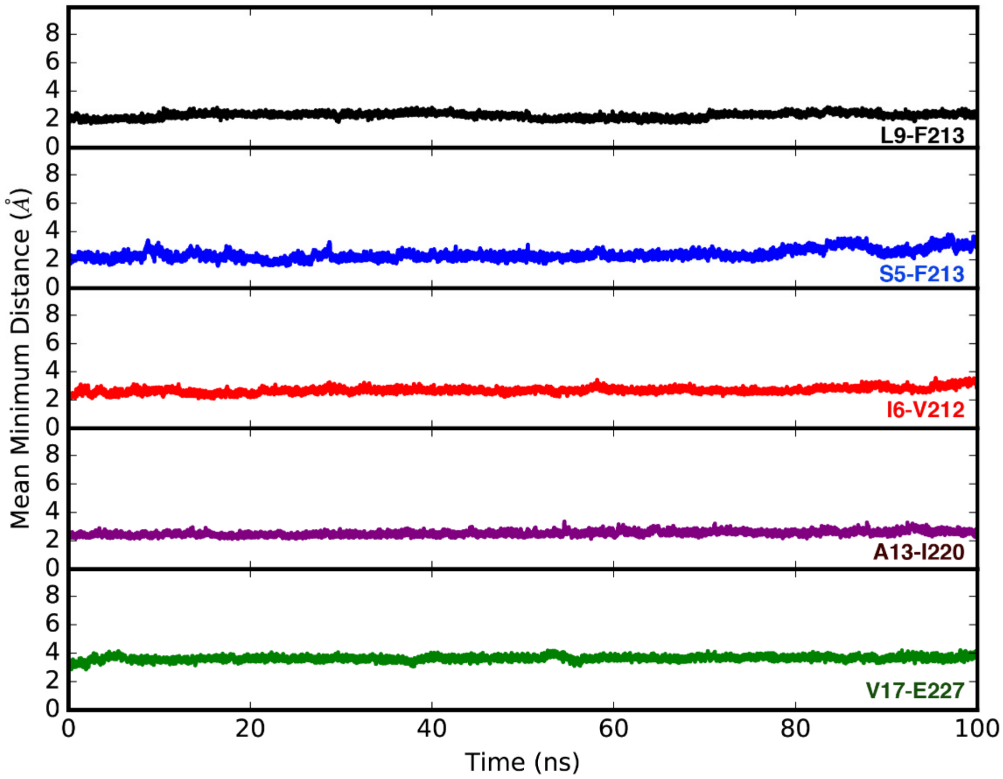

Figure S4. Molecular simulations of the interactions of TatA with TatC at its constitutive site and TatB with the TatC polar cluster site. TatA, TatB and TatC, assembled with TatB in the polar cluster site and TatA in its constitutive site, simulated for 100 ns as either **A.** Heterotrimeric $TatA_1B_1C_1$ or **B.** Heterononameric $TatA_3B_3C_3$. The plots show the mean minimum distance over time between the residues of TatA and TatC that are shown to be able to form cross-links. In both instances the residues remain in close proximity to one another, however, the $TatA_3B_3C_3$ complex appears to stabilise the overall motions of the individual subunits and therefore the distances between residues remain consistently low.

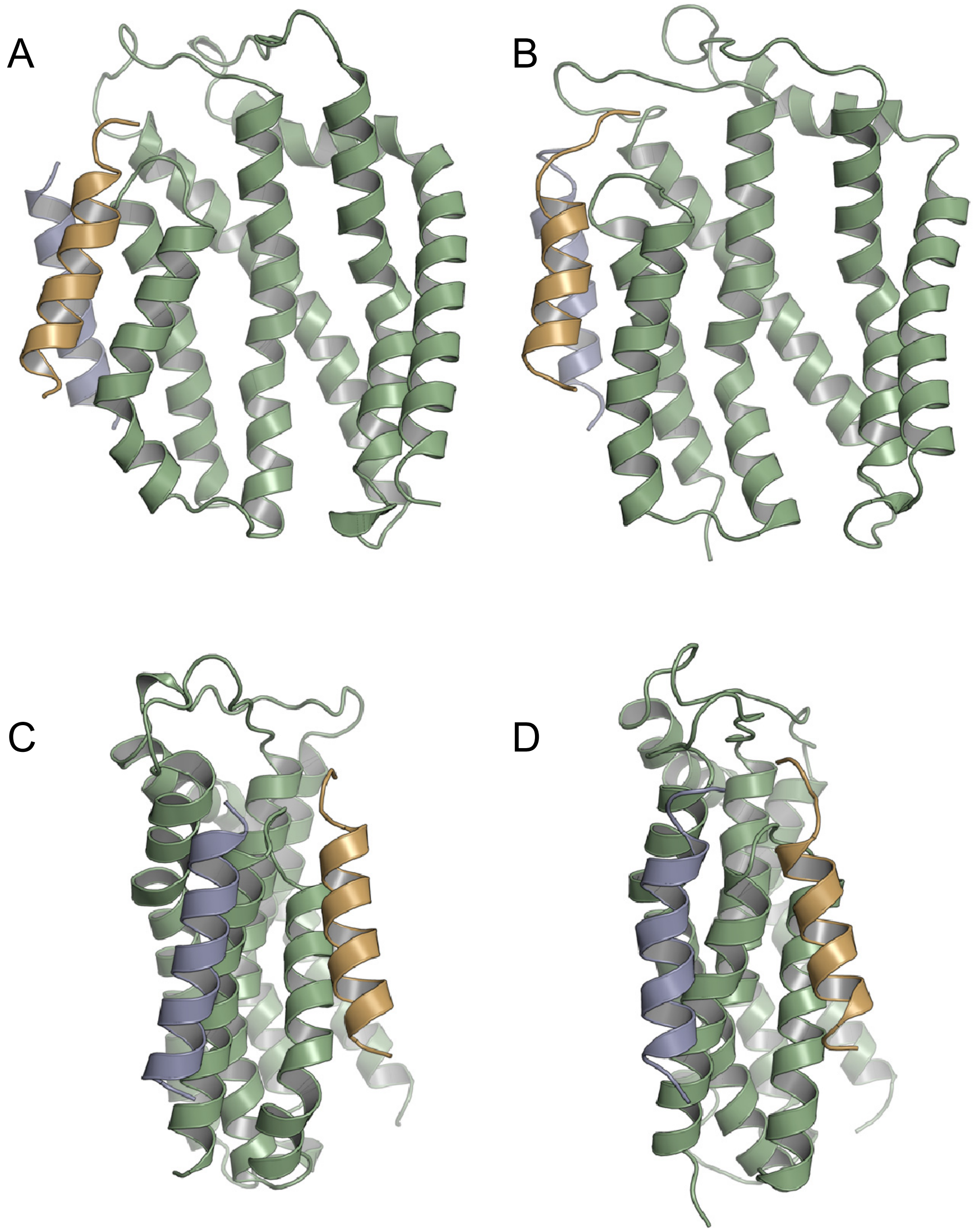

Figure S5. Illustrative snapshots of the molecular simulations of the TatABC complex under resting conditions, viewed from within the membrane, with TatB in the polar cluster binding site and TatA in the adjacent TM6 binding site of TatC. (A, C) 0 ns and (B, D) 100 ns. TatA is shown in silver-blue, TatB in gold and TatC in green.

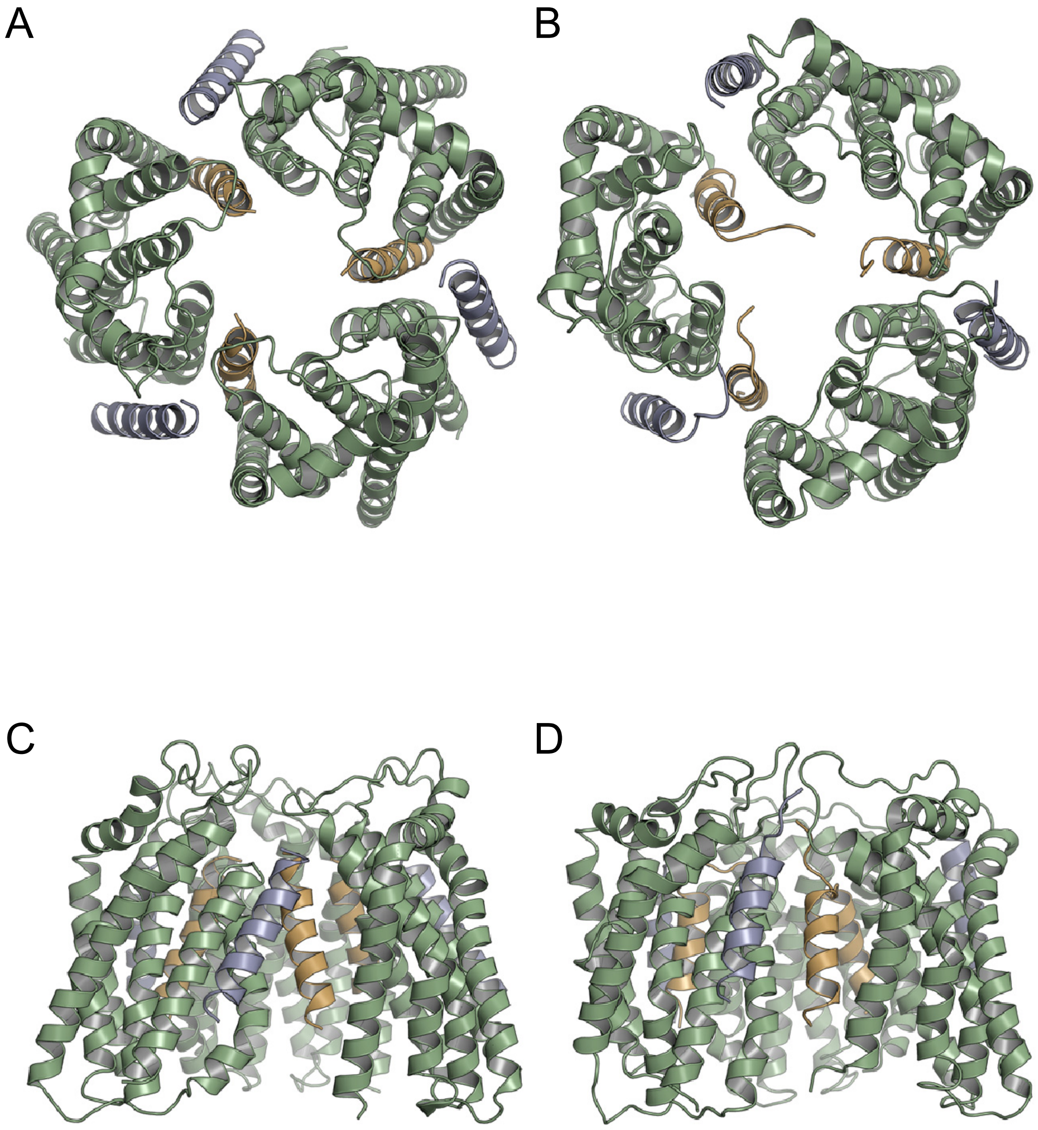

Figure S6. Illustrative snapshots of the molecular simulations of the trimeric TatABC complex under resting conditions with TatA in the polar cluster binding site and TatB in the adjacent TM6 binding site of TatC. (A, B) viewed from the periplasm and (C, D) within the membrane, at (A, C) 0 ns and (B, D) 100 ns. TatA is shown in silver-blue, TatB in gold and TatC in green.

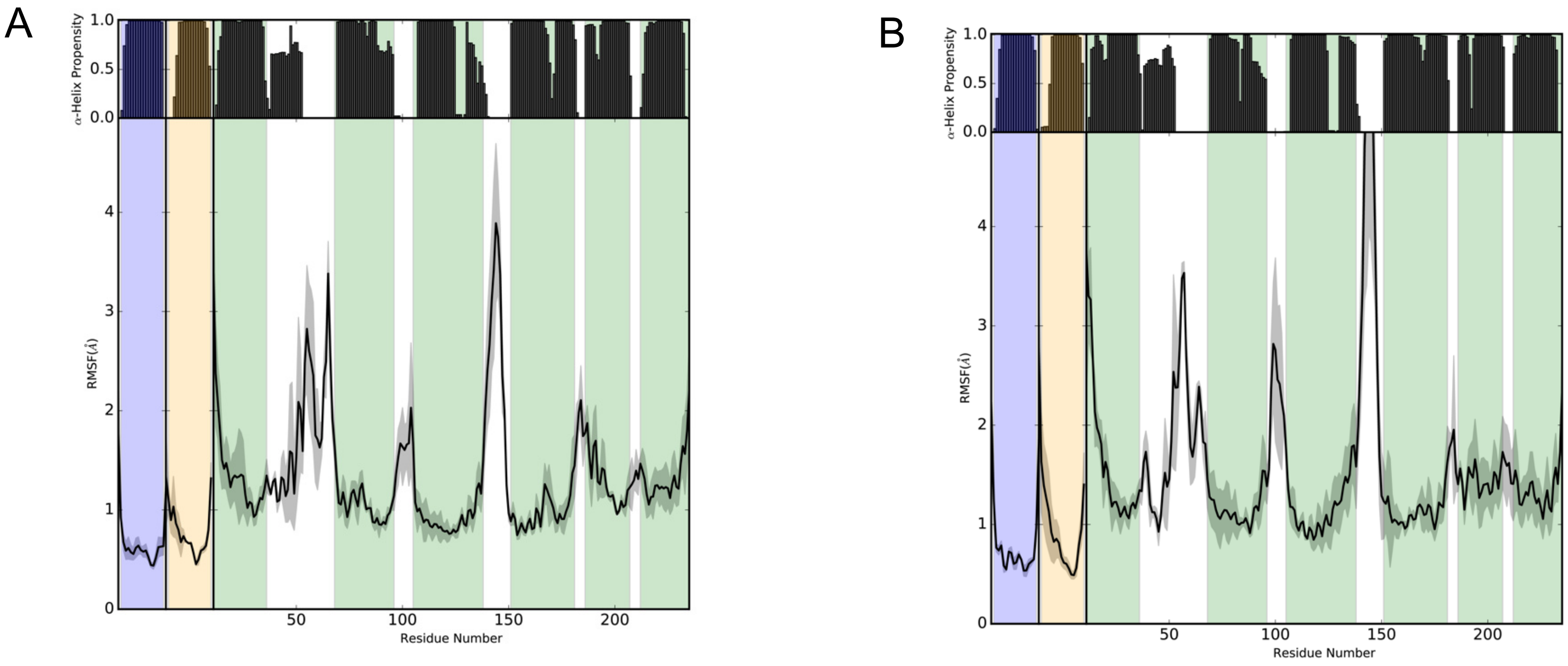

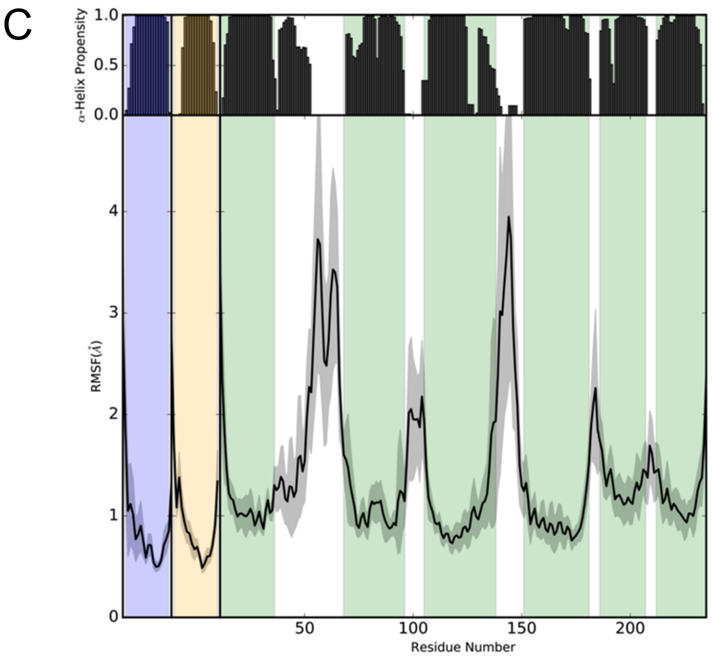

Figure S7. Structural stability plots for the modelled Tat protein complexes from molecular simulations. TatA, TatB and TatC, assembled with TatB in the polar cluster site and TatA in its constitutive site, for **A.** the heterotrimeric and **C.** heterononameric complexes. For **B.** TatA is in the polar cluster site and TatB in its constitutive site in a heterotrimeric TatABC complex**.** The plots illustrate the retention of α-helical secondary structure (black bars) for each molecular system, with the lower panels in each plot showing the residue fluctuations. α-helical regions of the plots are coloured blue for TatA, yellow for TatB and green for TatC.

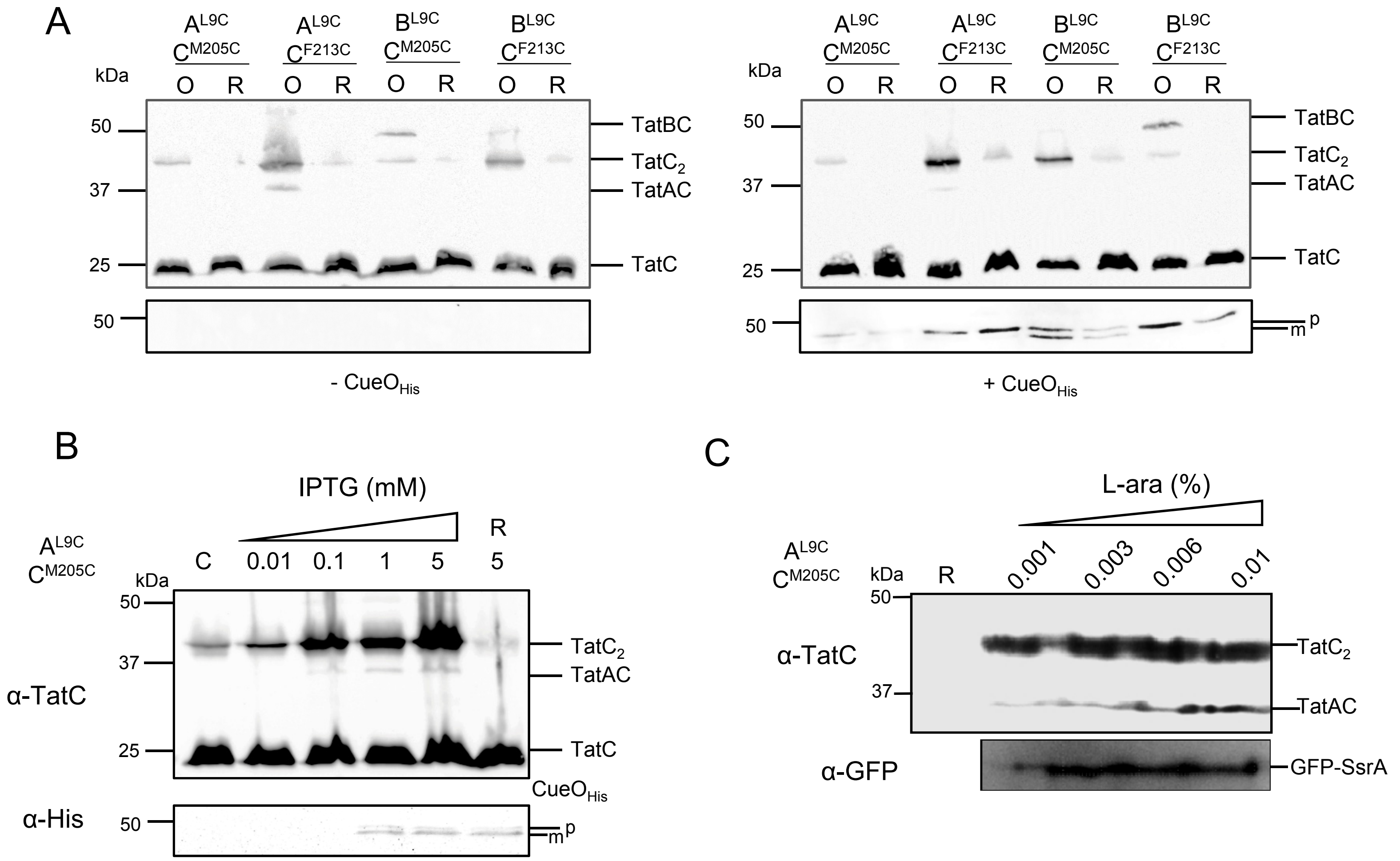

Figure S8. Overproduction of Tat substrates alter TatA and TatB crosslinking to TatC. A. Strain DADE harbouring plasmid p101CC4 producing either $TatA^{L9C}$ or $TatB^{L9C}$, alongside either $TatC^{M205C}$ or ^{F213C} and plasmid pQE80-CueO where indicated, was incubated in the presence of 1.8mM CuP for 1 min (O) or 10mM DTT for 1min (R). Following quenching, membrane fractions were immunoblotted with anti-TatC antiserum. **B.** Strain DADE harbouring plasmid p101CC4 producing $TatA^{L9C}$ alongside $TatC^{M205C}$ and plasmid pQE80-CueO, was left untreated (C) or incubated with IPTG concentrations between 0.01mM to 5mM as indicated. All samples were incubated in the presence of 1.8mM CuP for 1 min except for the reducing control (R), which was treated with 10mM DTT for 1min. Membrane fractions were immunoblotted with an anti-TatC antiserum. In A. and B. aliquots of the soluble fraction following membrane preparation were retained and analyzed by immunoblotting with an anti-Histag antibody. **C.** Strain DADE-P harbouring plasmids pUNITATCC4 producing $TatA^{L9C}$ and wildtype TatB alongside $TatC^{M205C}$ and pTGS encoding SsrA-tagged GFP fused to the TorA signal sequence was incubated in the presence of the indicated concentration of L-arabinose for 20 min before were addition of 1.8mM CuP (or 10mM DTT for sample marked R). Samples were incubated for 1 min, after which whole cells were analysed by immunoblotting with an anti-TatC antiserum. The bottom panel shows anti-GFP immunoblot of the same samples following arabinose addition but prior to incubation with CuP.

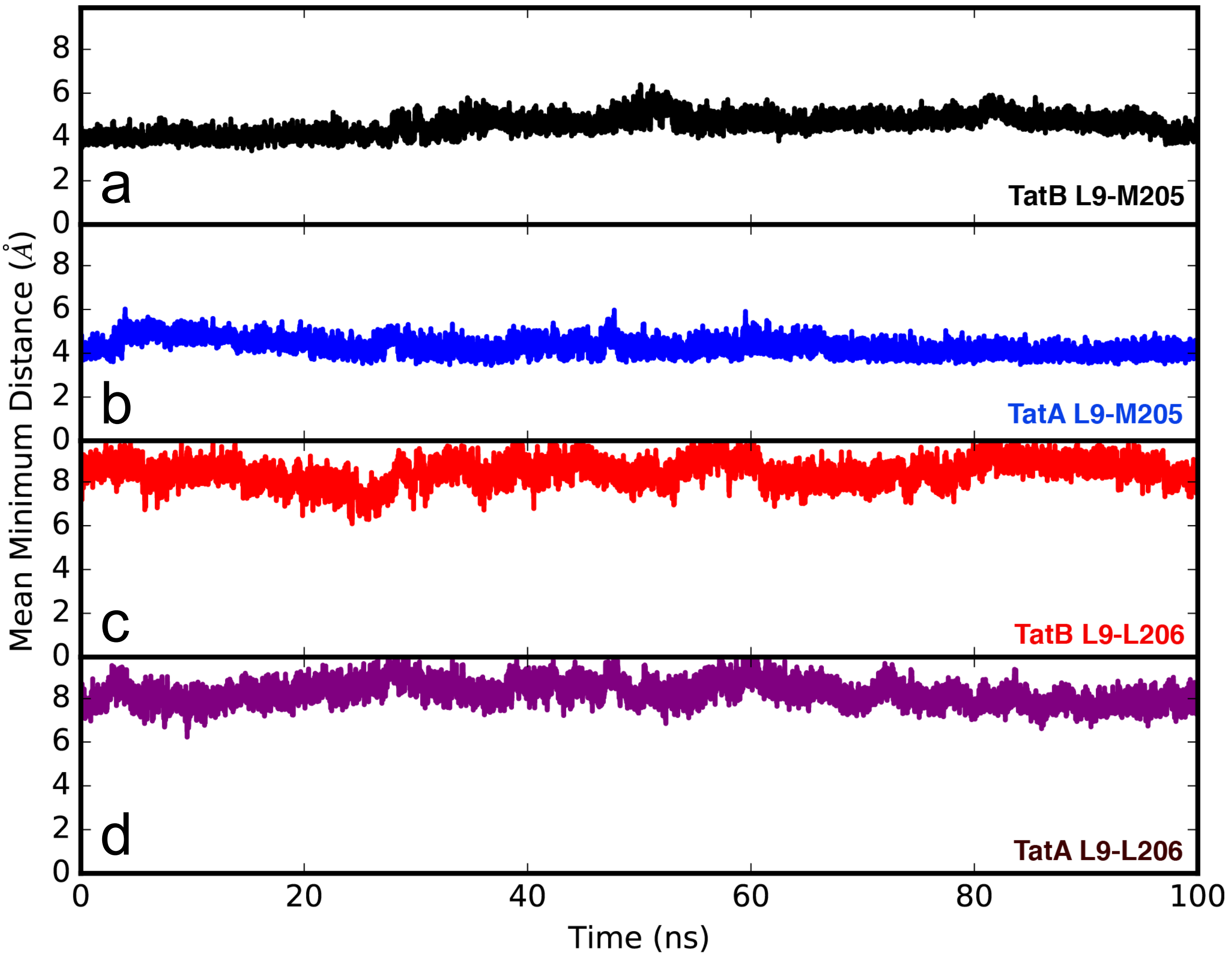

Figure S9. Molecular simulations of the interactions of TatA and TatB with each of the binding sites on TatC. A and **C.** TatA, TatB and TatC, assembled with TatB in the polar cluster site and TatA in its constitutive site for panels **B.** and **D.** TatA is in the polar cluster site and TatB in the constitutive site. Plots show the evolution of the minimum distances between L9 and either M205 or L206 during the 100 ns simulations. For both TatA and TatB, the simulations retain the close proximity between L9 and M205 at ~4 Å, while L9 to L206 is more variable, at around 8 Å.

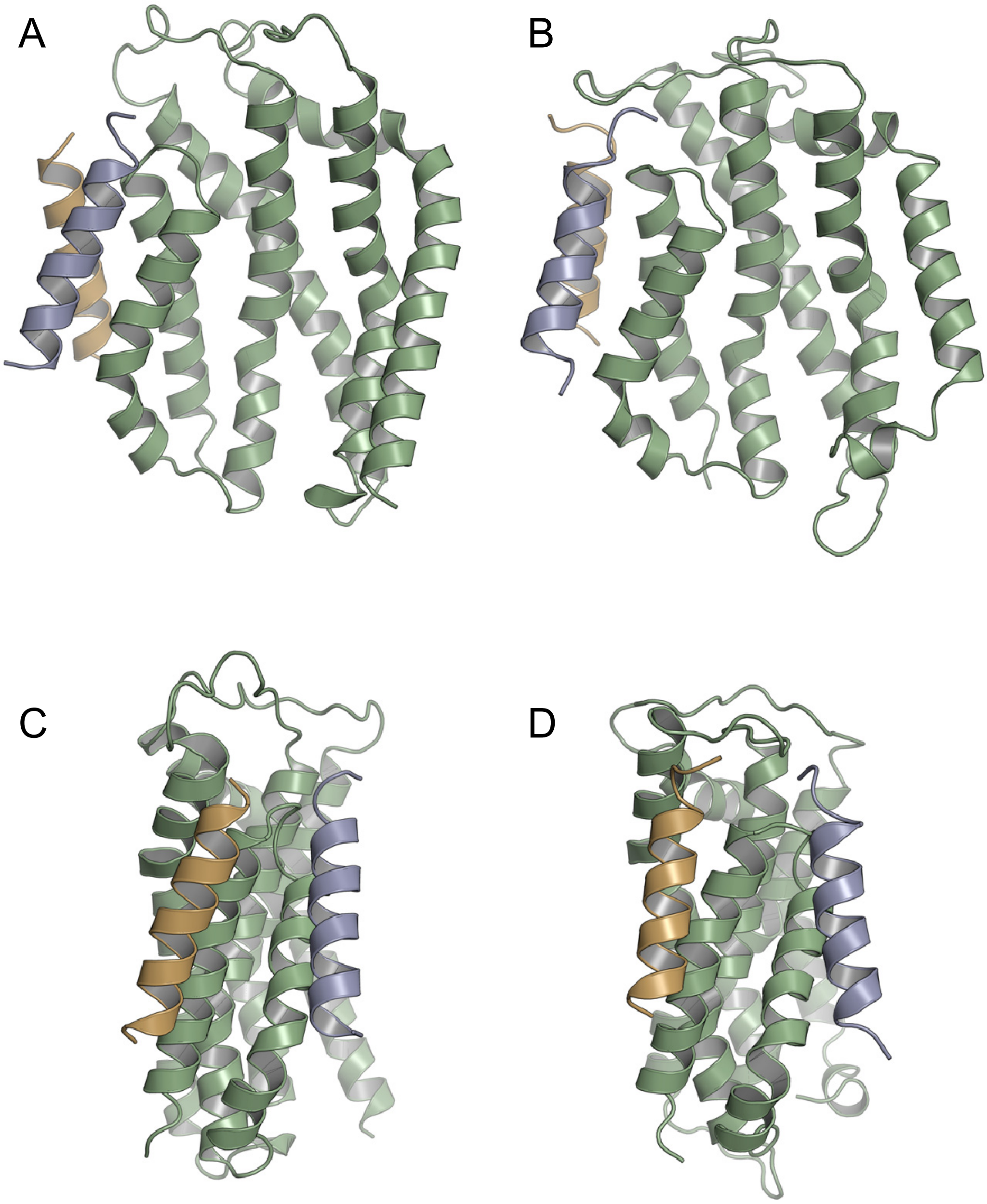

Figure S10. Illustrative snapshots of the molecular simulations of the TatABC complex upon substrate binding, viewed from within the membrane, with TatA in the polar cluster binding site and TatB in the adjacent TM6 binding site of TatC. (A, C) 0 ns and **(B, D)** 100 ns. TatA is shown in silver-blue, TatB in gold and TatC in green.
